# Unraveling historical introgression and resolving phylogenetic discord within *Catostomus* (Osteichthys: Catostomidae)

**DOI:** 10.1186/s12862-018-1197-y

**Published:** 2018-06-07

**Authors:** Max R. Bangs, Marlis R. Douglas, Steven M. Mussmann, Michael E. Douglas

**Affiliations:** 1 Department of Biological Sciences, University of Arkansas, Fayetteville, AR 72701 USA; 2 School of Fisheries, Aquaculture and Aquatic Sciences, Auburn University, Auburn, AL 36849 USA

**Keywords:** ddRAD, Hybridization, Introgression, Phylogenetic incongruence, Patterson’s D-statistic, *Catostomus*

## Abstract

**Background:**

Porous species boundaries can be a source of conflicting hypotheses, particularly when coupled with variable data and/or methodological approaches. Their impacts can often be magnified when non-model organisms with complex histories of reticulation are investigated. One such example is the genus *Catostomus* (Osteichthys, Catostomidae), a freshwater fish clade with conflicting morphological and mitochondrial phylogenies. The former is hypothesized as reflecting the presence of admixed genotypes within morphologically distinct lineages, whereas the latter is interpreted as the presence of distinct morphologies that emerged multiple times through convergent evolution. We tested these hypotheses using multiple methods, to including multispecies coalescent and concatenated approaches. Patterson’s D-statistic was applied to resolve potential discord, examine introgression, and test the putative hybrid origin of two species. We also applied naïve binning to explore potential effects of concatenation.

**Results:**

We employed 14,007 loci generated from ddRAD sequencing of 184 individuals to derive the first highly supported nuclear phylogeny for *Catostomus*. Our phylogenomic analyses largely agreed with a morphological interpretation,with the exception of the placement of *Xyrauchen texanus*, which differs from both morphological and mitochondrial phylogenies. Additionally, our evaluation of the putative hybrid species *C. columbianus* revealed a lack introgression and instead matched the mitochondrial phylogeny. Furthermore, D-statistic tests clarified all discrepancies based solely on mitochondrial data, with agreement among topologies derived from concatenation and multispecies coalescent approaches. Extensive historic introgression was detected across six species-pairs. Potential endemism in the Virgin and Little Colorado Rivers was also apparent, and the former genus *Pantosteus was* derived as monophyletic, save for *C. columbianus*.

**Conclusions:**

Complex reticulated histories detected herein support the hypothesis that introgression was responsible for conflicts that occurred within the mitochondrial phylogeny, and explains discrepancies found between it and previous morphological phylogenies. Additionally, the hybrid origin of *C. columbianus* was refuted, but with the caveat that more fine-grain sampling is still needed. Our diverse phylogenomic approaches provided largely concordant results, with naïve binning useful in exploring the single conflict. Considerable diversity was found within *Catostomus* across southwestern North America, with two drainages [Virgin River (UT) and Little Colorado River (AZ)] reflecting unique composition.

**Electronic supplementary material:**

The online version of this article (10.1186/s12862-018-1197-y) contains supplementary material, which is available to authorized users.

## Background

A principle goal of evolutionary biology is to derive relationships among living organisms, with a traditional assumption being a correspondence among gene trees and the species history [1]. However, recent phylogenomic studies have instead yielded gene trees that are largely, if not completely, incongruent [2, 3]. In this regard, evolutionary patterns are now recognized as more complex than originally perceived, and the large, multi-gene datasets that initially provoked these incongruences also possess the capacity for their deconstruction, which has now become a priority [4, 5]. Additionally, these complex evolutionary histories are now a mechanism that explains discrepancies with those derived using single gene approaches [6, 7].

Incongruence can often result from biological as well as methodological processes, and a good example would be how ancestral introgression has impacted the evolutionary history of a clade [8]. These issues were previously deemed rather inconsequential in that hybridization and introgression were regarded as not only rare but also with an inevitable end-result of ‘genetic swamping’ among parental taxa. Although the ‘rarity’ argument has long been rejected [9, 10], that of ‘genetic swamping’ has only recently come under scrutiny. For example, introgression occurs not only without the subsequent dismantling of species boundaries [11], but also with a rather precise transmission of adaptive traits [12, 13]. Consequently, a less myopic view of introgressive hybridization has now emerged, one that promotes the semipermeable nature of species boundaries, but also with rather specific consequences for genome evolution [14–16].

Phylogeographic studies have often relied on individual mitochondrial DNA genes excerpted from a single locus, a consideration that may not represent the complex evolutionary history of a study species [17]. This can be especially problematic with regards to mito-nuclear incongruence, particularly since mitochondrial genes are maternally inherited and prone to purifying selection [18]. In addition, Dobzhansky-Muller incompatibilities (i.e., the accumulation of incompatible epistatic interactions between diverging species), can lead to asymmetric introgression and a rapid fixation of heterospecific haplotypes, particularly when incompatibilities arise between the mitochondrial genes of one species and nuclear genes of a second, but not between the mitochondrial genes of the second and the nuclear genes of the first [19]. Consequently, the phylogenetic history of mitochondrial genes can differ substantially from those in the nuclear genome, and may conflict with the species tree. Such incompatibility has in fact been noted in numerous taxa: fruit flies [20], lizards [21], birds [22, 23], frogs [24, 25], mammals [26, 27], and fishes [28–30].

Fishes can be particularly problematic in this regard, due in large part to a natural history that facilitates hybridization, i.e., external fertilization, weak reproductive isolation, and a relatively linear dispersal in streams [31, 32]. It is a relatively conspicuous phenomenon in the genus *Catostomus,* commonly known as Finescale Suckers, because individuals hybridize readily when invasive congeners are introduced and/or habitats modified [33, 34]. Introgression may have been promoted in western North America by volcanism, glaciation, extreme flooding, and extended drought that, in turn, shifted distributions and abundances of species [35]. Long periods of vicariant-derived isolation were thus provided, and sporadically augmented by secondary contact due to stream capture [36, 37].

The evolutionary history of *Catostomus* has proven difficult to decipher, due largely to incongruent mitochondrial and morphological phylogenies. Two valid hypotheses have been proposed to explain these discrepancies: introgressive hybridization [35], and the convergent evolution of morphologies [38]. The former (i.e., the ‘Introgression Hypothesis’) offers an explanation for admixed genotypes in morphologically distinct lineages, as supported by several well-documented and contemporary hybridization events. The second (i.e., the ‘Convergent Evolution Hypothesis’) posits that mtDNA genealogies accurately reflect the species tree, but with distinct morphologies arising multiple times through convergent evolution, thus promoting an argument that “... the long-thought idea of widespread genetic exchange across taxa represents a series of declarations that are either less parsimonious or cannot be tested” [38].

Indeed it is difficult to separate introgression from incomplete lineage sorting, the latter defined as a situation in which alleles in one species share a more recent common ancestor with another due to random assortment of ancestral polymorphisms [39]. However, patterns of historical introgression have recently been deciphered through use of Patterson’s D-statistic [40], initially employed to test for hybridization among early hominid lineages [8], then subsequently applied to a variety of taxa: *Heliconius* butterflies [12], *Sceloporus* lizards [41], and *Xiphophorus* fishes [42]. The test necessitates thousands of loci that can be generated by various methods, to include cost-effective restriction-associated (RAD) DNA sequencing [12, 43, 44].

Here we apply one such method (i.e., double digest restriction-site associated DNA sequencing, or ddRAD; [45]) to: 1) test for the presence of introgression among *Catostomus* species and, 2) resolve the conflicts between their mitochondrial and morphological phylogenies. We employed different phylogenetic methods (i.e., concatenated SNPs and loci as well as multispecies coalescent) to assure that bias was not introduced by various algorithms used to resolve this complex evolutionary history. Any discordance between methods was also explored.

## Methods

### Sampling

**Fig. 1:**
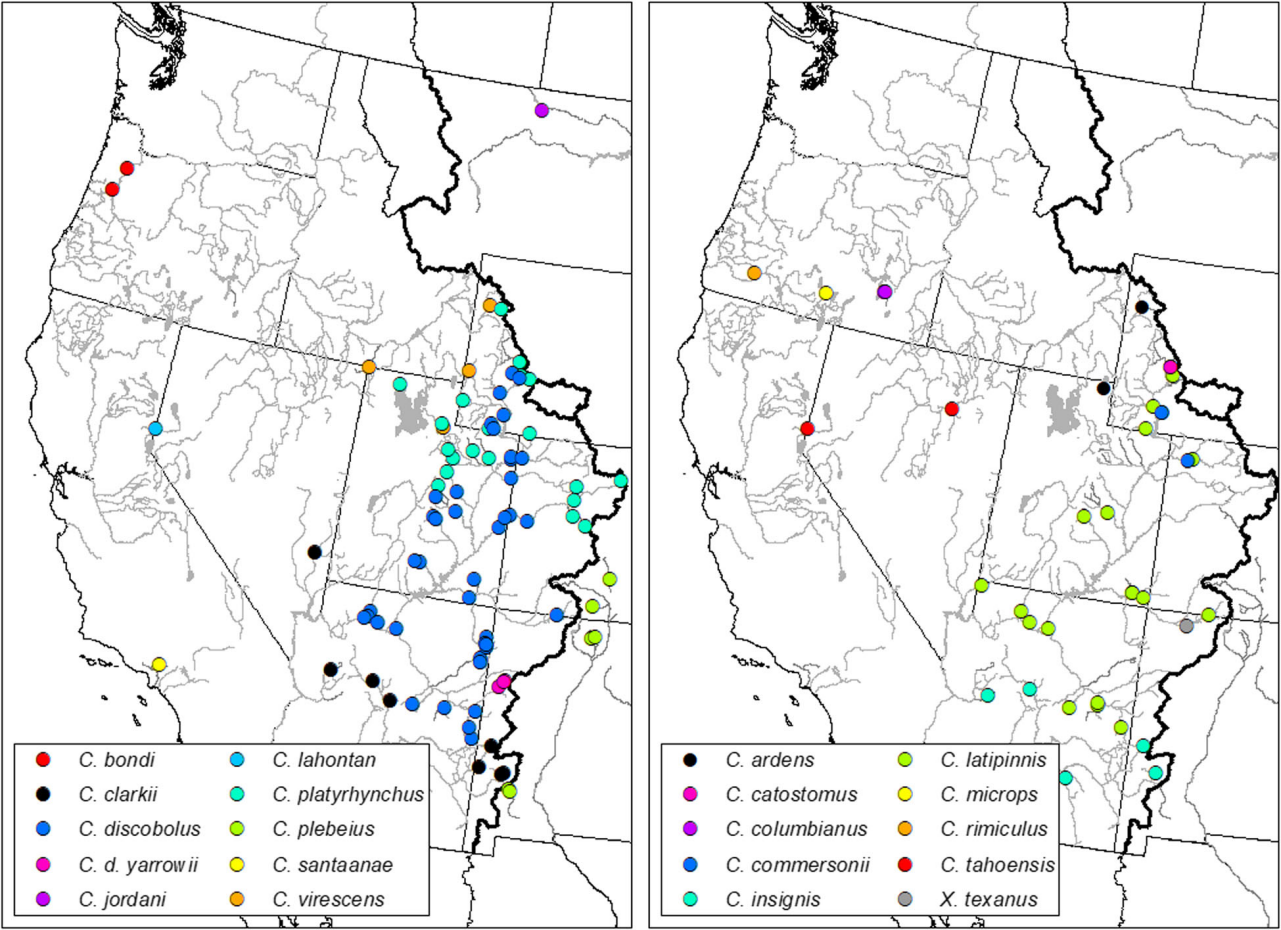
Map of sampling locations colored by species. Map split into two panels with the left panel containing members of the former *Pantosteus* and the second containing all other *Catostomus* and *Xyrauchen* samples

**Fig. 2:**
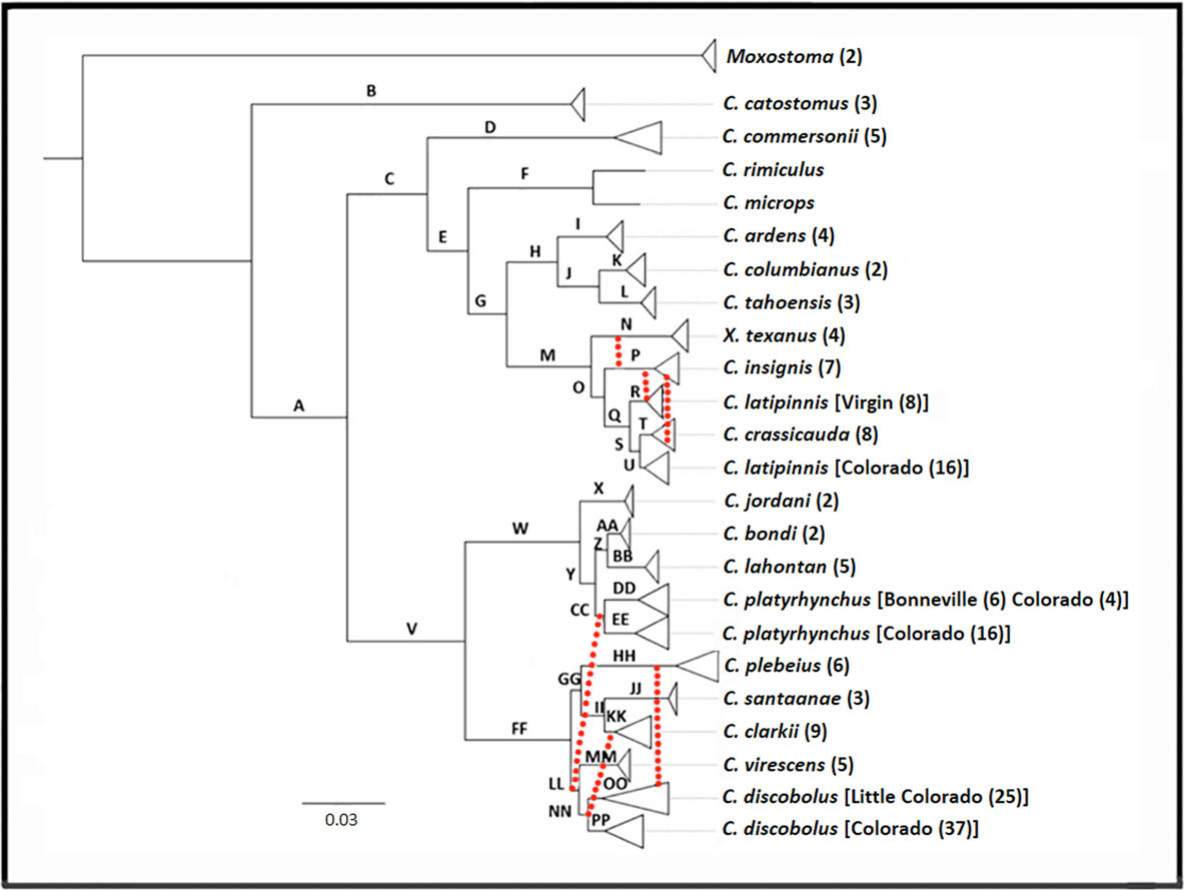
Phylogeny of *Catostomus* with branch lengths derived via RAXML. Letters at nodes correspond to columns in Fig. 3 and present support values for all analyses. Nodes are collapsed according to species and level of support. Those representing operational taxonomic units (OTUs) are discussed. Dotted lines represent significant introgression events per D-statistic tests. Numbers in parentheses represent individuals at each collapsed node

Our sampling included 20 species of *Catostomus* (180 samples). We also evaluated *Xyrauchen texanus* (four samples)*,* given its questionable phylogenetic placement within the family (Fig. 2, Table 1). Two species of *Moxostoma* (one sample each) were added as outgroup (Fig. 2, Table 1). The estimated divergence time of this genus (i.e., <50mya; [46]) places it within a temporal frame appropriate for ddRAD analyses [47, 49, 50]. Fin clips and tissue plugs were collected between 1995 and 2011, and spanned the range of focal taxa in western North America. Additional samples were obtained from the following museums: Ichthyology Collection, Oregon State University/ Corvallis; and Museum of Southwestern Biology, University of New Mexico/ Albuquerque (Table 1, see Acknowledgements for accession numbers). The diversity of species and their sampling locations permitted a rather fine-grained examination of phylogeographic patterns (Fig. 1).

**Table 1:** Drainage and State for *Catostomus*(=C), *Xyrauchen*(=X), *Moxostoma*(=M). Sites = Sample sites, N=Number of samples

Species	Major Drainage	State	Sites	N
*C. ardens*	Bonneville Basin	WY, UT	2	4
*C. latipinnis*	Upper Colorado River	WY, UT, CO, AZ, NM	11	11
	Grand Canyon	AZ	3	5
	Virgin River	UT	1	8
*C. “crassicauda”*	Little Colorado River	AZ	3	8
*C. insignis*	Lower Colorado River	AZ, NM	5	7
*C. jordani*	Missouri River	MT	1	2
*C. lahontan*	Lahontan Basin	NV	2	5
*C. bondi*	Columbia River	OR	1	2
*C. platyrhynchus*	Bonneville	WY, UT	4	6
	Upper Colorado River	WY, UT, CO	16	20
*C. virescens*	Bonneville Basin	WY, UT	5	5
*C. discobolus*	Upper Colorado River	WY, UT, CO, AZ, NM	29	31
	Grand Canyon	AZ	5	6
	Little Colorado River	AZ	8	13
*C. d. yarrowi*	Zuni River	NM	3	12
*C. clarkii*	Virgin River	NV	1	1
	Lower Colorado River	AZ, NM	7	8
*C. santaanae*	Los Angeles River	CA	1	3
*C. plebeius*	Rio Grande	CO, NM	6	6
*C. commersonii*	Mississippi River	ND, IL	3	3
	Upper Colorado River	WY, CO	2	2
*C. tahoensis*	Lahontan Basin	NV	1	3
*C. rimiculus*	Rogue River	OR	1	1
*C. microps*	Goose Lake	OR	1	1
*C. columbianus*	Donner und Blitzen River	OR	2	2
*C. catostomus*	Upper Colorado River	WY	1	3
*X. texanus*	Upper Colorado River	UT, NM	2	4
*M. macrolepidotum*	Mississippi River	ND	1	1
*M. valenciennesi*	Mississippi River	MN	1	1
		Total	129	184

### Data collection

Genomic DNA was extracted from tissue samples using the PureGene® Purification Kit or DNeasy® Tissue Kit (Qiagen Inc.) and stored in DNA hydrating solution. The quantity and quality of high molecular weight DNA were visualized on 2% agarose gels and quantified using a Qubit fluorometer (Thermo Fisher Scientific, Inc.). Library development followed previously published protocols (Peterson et al. 2012). Digests were performed using 1 μg of genomic DNA with 10 units each of PstI (5’-CTGCAG-3′) and MspI (5’-CCGG-3′) in CutSmart buffer (New England Biosciences) for 20 h at 37 °C. Digests were visualized on 2% agarose gels, cleaned using AMPure XP beads, and quantified using a Qubit fluorometer. Approximately 0.1 μg of DNA was then ligated with barcoded Illumina adaptors, using custom oligos [45]. All barcodes differed by at least two bases so as to avoid fragment mis-assignments.

Ligations were pooled in sets of 48, cleaned and concentrated using AMPure XP beads, then size selected at 350–400 bps using the Pippin Prep automated size fractionator (Sage Sciences). Size-selected DNA served as template for Phusion high-fidelity DNA polymerase reactions using indexed primers and 10 cycles, following the manufacture’s protocol (New England Biosciences). Reactions were cleaned with AMPure XP beads, and visualized on the Agilent 2200 TapeStation to confirm successful amplification. A final quality check of libraries was performed via qPCR at the University of Wisconsin Biotechnology Center (Madison), and two index libraries (96 samples) were pooled per lane for Illumina HiSeq 2000 100-bp single-end sequencing.

### Filtering and alignment

All analyses were conducted on the Arkansas High Performance Computing Cluster (AHPCC) at University of Arkansas. Illumina reads were filtered and aligned using the pipeline PyRAD v.3.0.5 [6]. Restriction site sequences and barcodes were removed, resulting in 87 bp-fragments. Loci were discarded if they exhibited: 1) < 5 reads within an individual; 2) > 10 heterozygous sites per individual consensus, 3) > 2 haplotypes per individual; 4) > 75% heterozygosity per site among individuals; and 5) < 50% of individuals within a given locus (per [49]). Individuals with more than 80% missing data were also discarded.

Selecting the appropriate clustering threshold is problematic especially with regard to gene duplications [41]. Importantly, at least four whole genome duplications (WGD) have occurred in *Catostomus:* Two at the base of all vertebrates [50], one at the base of all teleosts (~350mya-320mya; [51, 52]), and one at the base of the family Catostomidae [53]. The date for the last event is relatively ambiguous, due to conflicted age-estimates for Catostomidae. This event was first approximated it at >50mya [46], but recent fossil calibrations involving mitochondrial genes suggest an older origin [54]. Thus, our best estimate for polyploidization (at least 50mya) is thus outside of the recovery range of RAD-seq methods, based on gain and loss of restriction sites [47, 48]. In addition to standard filters for paralogs (filters 2 through 4 above), we also tested clustering thresholds from 60 to 95% at 5% intervals, and subsequently evaluated how these affected our analyses. The premise for this approach was the following: Paralogs will cluster together at lower thresholds, with the potential of passing through our filtering at a level high enough to bias results. If so, then different topologies would be expected as clustering thresholds varied. However, we found instead that all clustering thresholds yielded the same concatenated topology, with differences only in support values. We then employed a clustering threshold of 80%, as derived from the uncorrected sequence divergence (per [55]) in four nuclear loci evaluated across the breadth of catostomid fishes [38, 56].

### Phylogenetic methods

Loci produced in PyRAD were used to generate phylogenies based on concatenated data. This included a maximum likelihood (ML) phylogeny (RAxML v. 7.3.2; [56]), using GTRCAT with 1000 bootstraps, and a Bayesian (BA) phylogeny (MrBayes v. 3.2.3; [57]) using GTR with 10 million generations sampled every 1000, with the first 25% discarded as burn-in. The larger datasets provoked computational limits in each program, necessitating the use of concatenated SNPs as input. Intact loci were also evaluated using GTRCAT with 1000 bootstraps (ExaML; [58]) so as to assess any potential impacts that result from the use of SNPs to derive our ML phylogeny. Heterozygosity was maintained in both SNP and whole loci alignments (coded by PyRAD following the IUPAC ambiguity codes), as well as insertions/deletions.

Values associated with poorly supported or erroneous nodes can be inflated when concatenated data are employed [59, 60]. This is especially problematic with regards to introgression, and can potentially result in a topology that is unsupported by the majority of loci [61, 62]. Since potential introgression has ensued between several species of *Catostomus*, we employed two multispecies coalescent analyses (MSC) suitable for RAD loci [49]. One of these (i.e., SVDquartets; [63], as implemented in Paup* v. 4.0 [64]), utilizes one SNP per RAD-locus with subsequent frequencies for each species used to test support for quartets. Alignments for SVDquartets contain one SNP per RAD-locus for each individual chosen by PyRAD with heterozygosity as previously coded. SNP frequencies for each species were then determined in SVDquartets by pooling individuals based on a priori partitioning into species (or populations) as derived from the concordance between taxonomic hypotheses, geographic distributions, and elevated support values in phylogenetic analyses of concatenated data. All possible quartets were sampled and bootstrapped (*N* = 1000).

The second MSC method (implemented in Astral v.4.7.8; [65]) constructs RAxML phylogenies using whole RAD loci, and then assesses support within these phylogenies using quartets. However, the small size of the RAD loci (87 bps) may in turn yield poor support. To adjust for this, a naïve binning method [66] was used to randomly group RAD loci into larger “supergenes” for input. To adjudicate any potential bias due to concatenation, the binning process combined loci into groups of 1, 2, 3, 5, 10, 20, 50, and 100. Nevertheless, tradeoffs are still apparent in that less bias can occur with lower binning levels but also less potential resolution, whereas higher resolution with greater bias stem from elevated binning levels (similar to methods based on concatenation). We did not filter for levels of polymorphism in these runs, which may reduce resolution at low levels of binning. Yet, filtering for polymorphic loci can also bias the phylogeny as well. To accommodate, we also reran our Astral analyses but with additional filtering to remove loci with < 2 polymorphic sites, or those with insertion-deletions. We also bootstrapped these runs (*n* = 128) using gene and site resampling, with results reported as percentages (Perl script: https://github.com/smussmann82/astral_pipeline).

### Patterson’s D-statistic

We tested resulting phylogenetic hypotheses for potential introgression events by gauging reproductive isolation with subsequent gene flow using Patterson’s D-statistic [40], as implemented in PyRAD. Given the phylogenetic conflicts between morphological [35] and mitochondrial [38] analyses, we examined the following pairs of species: *C. discobolus* x *C. platyrhynchus*, *C. discobolus* x *C. clarkii*, *C. discobolus* x *C. plebeius*, *C. latipinnis* x *C. insignis*, *C. insignis* x *X. texanus*, and *C. latipinnis* x *X. texanus*. The putative hybrid origin of *C. columbianus* [35] was also gauged.

All members of a given species were used in each D-statistic test, and all combinations were permuted so as to provide multiple tests per introgression event. Z-scores for individual permutations were derived from 1000 bootstrapped calculations (per [6]). Significance thresholds were adjusted for multiple tests using the Holm-Bonferroni correction, with α = 0.01 (per [67]). In cases where variance in D-statistic scores were elevated between populations within a species, we split populations according to geographic regions, so as to more appropriately gauge fine-grained patterns of introgression that may have impacted some populations more so than others.

We applied Partitioned D [6] and D FOIL tests [68] in an attempt to determine the potential direction of gene flow. However, the extended number of categories in these tests and their accuracy prevented any comparisons that contained zero-values as a component of the ratios used. These tests require a large number of loci with SNP variants that are diagnostic within each of the species, as well as random SNPs that unite each species pair. This was difficult to achieve given the gain and loss of loci in ddRAD, and particularly so across distantly related species [48]. In addition, the required sequencing effort was beyond the scope of this study. Although this limits our ability to assess directionality of gene flow, it does not limit our capacity to ascertain introgression nor does it constrain our capacity to recognize and explain the presence of discordance.

## Results

After filtering, 14,007 loci containing 179,811 SNPs were obtained, of which 67.9% (*N* = 122,128) were parsimoniously informative, with 32.68% missing values in the loci by individual matrix (Additional file 1: Figure S1, Additional file 2: Table S1). The aligned length of all concatenated loci was 1,337,556 bp, with 8.9% containing gaps. Average number of variable sites per locus was 12.8, ranging from none (18 loci) to a maximum of 46 (one locus). These data also produced 13,989 unlinked SNPs, where one SNP per RAD-locus was chosen for all loci containing at least one SNP (via the unlinked SNP output option in PyRAD). Average post-filtering coverage was 17.3×, and all individuals (*N* = 184) had > 8.6× coverage. Most samples (82%) contained 10–30% missing data, and those with greater amounts were randomly distributed across operational taxonomic units (OTUs), the exception being those from the Oregon State University museum at 40–80%. Missing values also showed some relationship with phylogenetic placement, consistent with the gain and loss of restriction sites (Additional file 1: Figure S1) (per [48]).

### Phylogenetic analyses

**Fig. 3:**
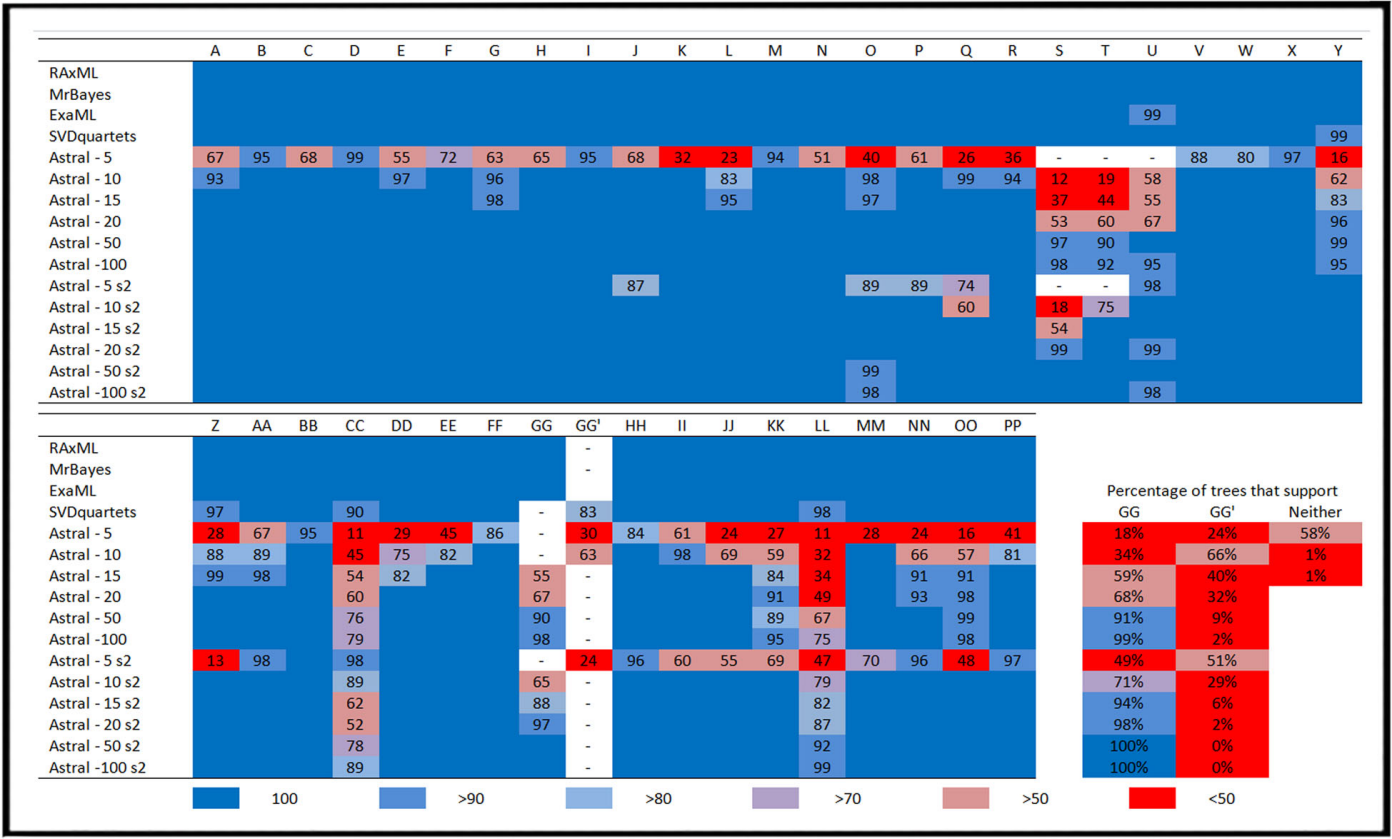
Nodal support values for all phylogenetic methods. Numbers to right of Astral = number of loci binned for each run where s2 = filtered data. Column headers = nodes in Fig. 2. Numbers below column headers = bootstrap support. Blue boxes with no values = 100% bootstrap support (1.0 posterior probability). Cell color: Blue = higher support, red = lower support, white(−) = no support, with cell colors varying from blue to red. Trees that supported individual binned loci for each Astral run are presented as colored cells at lower right corner of table

Both ML and BA methods generated from concatenated SNPs produced similar topologies, with OTU support at 100%. However, support within-OTUs varied, indicating somewhat less distinct and finer-grained phylogeographic patterns. ML results for whole concatenated loci (ExaML) matched that of concatenated SNP methods, with > 98% bootstrap support among OTUs (Figs. 2 and 3).

*Catostomus catostomus* was sister to all in-group species (Fig. 2), whereas the remaining *Catostomus* (group A) split into two large groups, one of which represented the former genus *Pantosteus* (group V) save *C. columbianus*, which was sister to *C. tahoensis* (group J) in the second group (group C) comprising the remainder of *Catostomus* external to *Pantosteus*.

Within the *Pantosteus* group (Fig. 2: group V), two distinct sister groups were identified, one corresponding to the ‘*platyrhynchus*’ (group W), and containing four recently described or re-designated species [35]. These are: *C. jordani* (Missouri River Basin), *C. bondi* (Columbia River Basin), *C. lahontan* (Lahontan Basin), and two groups of *C. platyrhynchus* (Upper Snake River/ Bonneville/ Colorado River basins) (groups DD and EE).

The remainder of *Pantosteus* (i.e., ‘*discobolus group*’; group FF) clustered into six components, three of which were previously described from the Upper Colorado River Basin (*C. discobolus*; group PP), and the Upper Snake River/Bonneville Basin (*C. virescens*; group MM), as well as an undescribed group (OO) that included *C. d. yarrowi* as well as *C. discobolus* from the Little Colorado River. The remaining three components represented *C. plebeius* (group HH), *C. santaanae* (group JJ), and *C. clarkii* (group KK).

**Fig. 4:**
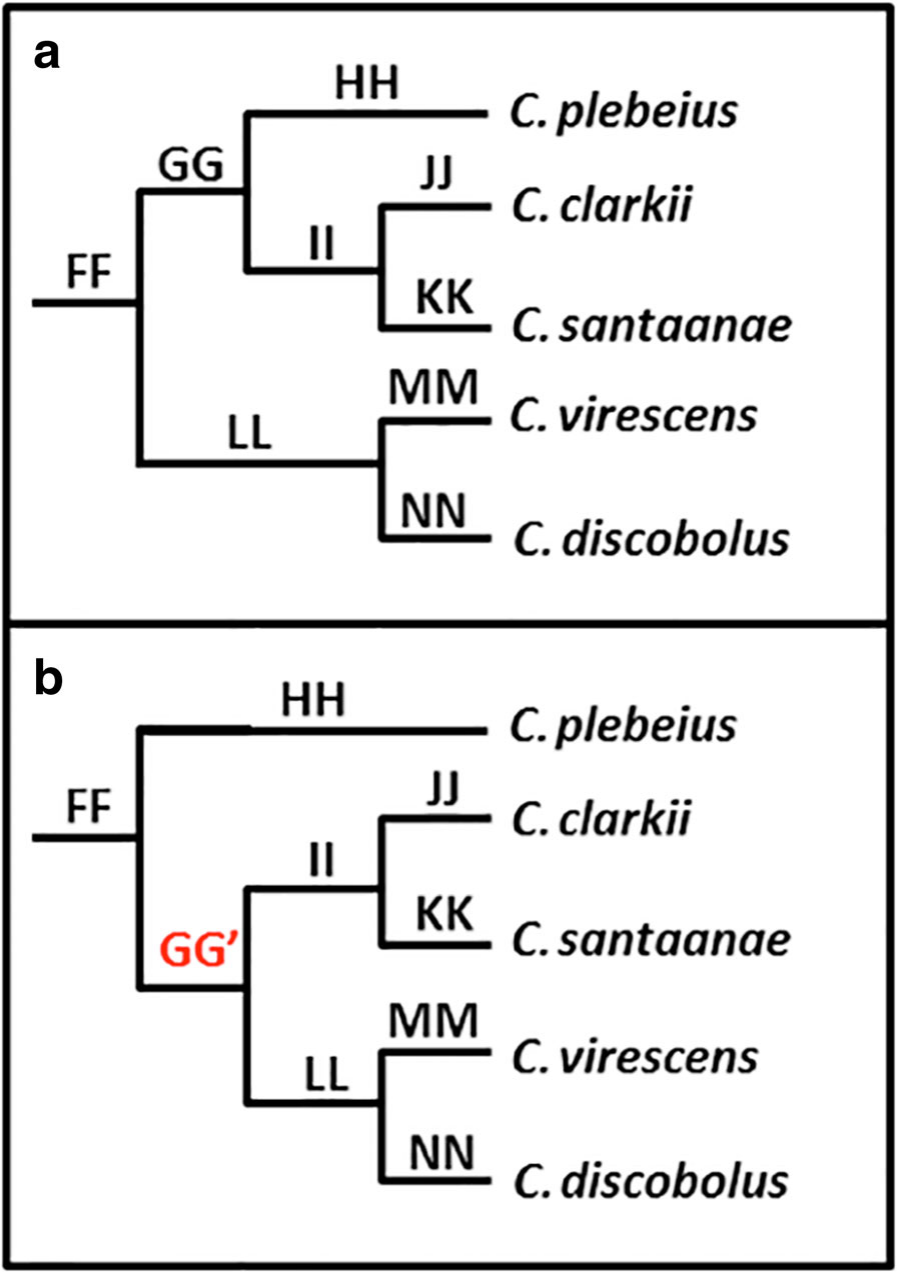
Alternative phylogenetic hypotheses for taxa in ‘*discobolus*’ group*,* as derived by (**a**) concatenated SNP approaches (RAXML, MRBAYES), and (**b**) multispecies coalescent approach (SVDQUARTETS). Letters at nodes correspond with columns in Fig. 3 that contain support values for all analyzes

SVDquartets (an MSC method) produced a topology similar to those from the concatenated SNP methods, but differed in placement of taxa within the ‘*discobolus*’ group (FF; Fig. 2). The SVDquartets analysis placed *C. plebeius* as external to this group (GG’; Fig. 4b), whereas the concatenated methods did so with both *C. discobolus* and *C. virescens* (GG; Fig. 4a).

Binning of < 5 RAD loci in the Astral analysis yielded little or no nodal support, and values are thus not reported. When binning included 5–10 RAD loci, the topology matched that of other MSC methods, with *P. plebeius* at the base of the ‘*discobolus*’ group (GG’; Fig. 4b). With binning of > 10 RAD loci, nodal support was generally higher and the topology reflected that from the concatenated methods, with *C. discobolus* and *C. virescens* at the base of the ‘*discobolus*’ group (GG; Fig. 4a, Fig. 3). When loci with fewer than two variant sites were filtered, the results largely matched that of the first set of runs, but with higher overall support. Filtered runs with reduced binning (5 RAD loci) matched that of MSC methods while higher binning runs (> 5 RAD loci) matched that of concatenated methods (Fig. 3), thus reflecting results from the first set of Astral analyses.

### Phylogenetic discordance and introgression

A summary of D-statistic test results can be found in Table 2, with a complete listing provided in Additional file 3: Table S2. Tests for introgression in *C. columbianus*, a species suggested to be of putative hybrid origin, were not significant despite the inclusion of several potentially co-occurring former *Pantosteus* species: *C. virescens*, *C. bondi*, *C. lahontan*, and the two groups of *C. platyrhynchus*.

**Table 2:** Results from Patterson’s D-statistic analyses

P1	P2	P3	O	D	BABA	ABBA	nloci	RangeZ	nSig/ntest
*tahoensis*	*columbianus*	*Pantosteus*	*catostomus*	0.20	6	9	1155	0, 2.97	0/324
*latipinnis* (UCR)	***latipinnis*** **(VR)**	***insignis***	*commersonii*	**0.64**	20	91	4655	**4.74, 12.69**	**840/840**
*latipinnis* (UCR)	***latipinnis*** **(WEN)**	***insignis***	*commersonii*	**0.48**	17	50	3169	0.21, **7.43**	**255/360**
*latipinnis*	***insignis***	***texanus***	*commersonii*	**0.53**	37	121	3484	2.73, **11.00**	**1039/1080**
*latipinnis* (UCR)	*latipinnis* (VR)	*texanus*	*commersonii*	0.44	17	43	4293	1.04, **6.01**	112/336
*virescens*	***discobolus*** **(UCR)**	***clarkii***	*jordani*	**0.62**	38	162	3540	**4.54, 18.54**	**1650/1650**
*virescens*	***discobolus*** **(ULCR)**	***clarkii***	*jordani*	**0.40**	48	112	3449	2.53, **7.53**	**767/1000**
*virescens*	*discobolus* (WAS)	*clarkii*	*jordani*	0.13	47	61	2619	0.12, 2.96	0/200
*santaanae*	***clarkii*** **(VR)**	***discobolus***	*jordani*	**0.43**	35	86	3463	**3.96, 7.08**	**66/66**
*santaanae*	*clarkii* (BW)	*discobolus*	*jordani*	0.18	22	31	2108	0.12, 2.65	0/132
*santaanae*	*clarkii* (GI)	*discobolus*	*jordani*	0.24	21	35	2879	0.17, 3.13	0/330
*plebeius*	***virescens***	***platyrhynchus***	*catostomus*	**0.38**	22	50	2425	2.22, **5.63**	**818/1080**
*plebeius*	***discobolus***	***platyrhynchus***	*catostomus*	**0.46**	20	54	2548	2.32, **9.28**	**2022/2376**
*jordani*	***platyrhynchus***	***virescens***	*catostomus*	**0.40**	24	57	2236	2.71, **9.65**	**327/360**
*jordani*	***platyrhynchus***	***discobolus***	*catostomus*	**0.41**	26	62	2567	2.21, **11.72**	**669/720**
*virescens*	*discobolus*	*platyrhynchus*	*catostomus*	0.02	23	25	2898	0, 2.41	0/1980
*discobolus*	***d. yarrowi*** **(RNU)**	***plebeius***	*jordani*	**0.78**	19	152	2932	**4.35, 36.52**	**864/864**
*discobolus*	*d. yarrowi* (AGR)	*plebeius*	*jordani*	0.11	14	17	1772	0.14, 1.17	0/128
*discobolus*	*d. yarrowi* (TAM)	*plebeius*	*jordani*	0.09	17	21	2012	0.01, 0.91	0/128
*clarkii*	*santaanae*	*plebeius*	*jordani*	0.14	18	24	2280	0, 3.52	0/288

Taxa used in comparisons include two sister taxa (=P1, P2), one taxon outside of P1, P2 (=P3), and one outgroup (=O). Positive D-statistics (=D) represent an excess of loci supporting ABBA verses BABA topologies, thus indicating potential introgression between taxa P2 and P3. Range of Z-scores for each set of tests (=RangeZ) and the number of significant tests out of the total number of tests (=nSig/ntest) are also reported, as is the overall Z-score (=Z), average number of alternatively discordant loci (=BABA and ABBA), and the average number of loci per test (=nloci). Significant Z-scores are in bold, as are the species involved with introgression. All tests are represented by species names with the exception of *Pantosteus* where multiple species occur in the former genus. Some species are divided into region, with abbreviations as follows: *UCR* Upper Colorado River, *TAM* Tampico Springs, *AGR* Agra Remora, *RNU* Rio Nutria, *ULC* Upper Little Colorado, *WAS* Willow and Silver creeks, *WEN* Wenima Wildlife Area, *VR* Virgin River *BW* Bill Williams River, *GI* Gila River Basin

For the remainder of the tests, it was important to separate groups that are phylogeographically distinct, since the presence or magnitude of introgression may differ for each and could be masked if all were clumped into a single group. We split several species due to their elevated within-species variances: *C. platyrhynchus* was divided between Bonneville/Snake and Colorado rivers, whereas *C. clarkii* was broken into three (i.e., Virgin, Bill Williams, and Gila rivers), all of which were supported at 100% in all concatenated phylogenetic methods. Our phylogenetic results also supported the split of *C. latipinnis* into the same three groups (i.e., Virgin, Little Colorado, and Colorado rivers), with additional separation into Grand Canyon and the remainder of the Upper Colorado River. Samples from Wenima Wildlife Area (AZ) had substantially different D-statistic values and were thus split from the Little Colorado River. Finally, we separated *C. discobolus* into groups similar to those for *C. latipinnis* (i.e., Grand Canyon, Little Colorado, and Upper Colorado rivers), with an additional split to accommodate the presence of heterospecific alleles in the Little Colorado River [69].

Introgression between *C. latipinnis* and *C. insignis* was noted at two sites (Virgin River and Wenima Wildlife Area), with but two individuals (67%) significant in the latter. Evidence was also detected for introgression between *X. texanus* and *C. insignis*, but not between *C. latipinnis* and *X. texanus*.

Introgression was also detected between *C. clarkii* and *C. discobolus*, but with considerable variance in the D-statistic that underscored a geographic pattern among sites. In *C. discobolus*, all Colorado River basin groups were introgressed, save for two sites in the Little Colorado River drainage (i.e., Willow and Silver creeks) that lacked statistically significant D-statistics. The D-statistic was also greater for sites in the Upper Colorado River Basin above Lake Powell (AZ/UT border) than for Grand Canyon and the Little Colorado River, its major tributary in the Lower Basin (Additional file 3: Table S2). No statistically significant introgression was detected in *C. clarkii*, save for a single Virgin River sample.

Introgression was also detected between *C. discobolus* and *C. platyrhynchus* in the Colorado River, as well as between *C. virescens* and *C. platyrhynchus* in the Upper Snake/ Bonneville basins. However, no geographic pattern of introgression was apparent when these species were compared within and between basins (Additional file 3: Table S2).

Introgression of *C. plebeius* into *C. discobolus* was not statistically significant, save for a single population in the Rio Nutria of the Zuni River, NM (a tributary of the Little Colorado River). Other Zuni River populations (i.e., Agua Remora and Tampico Springs), and the remainder of the Little Colorado River, lacked statistical significance. Similarly, no introgression was detected among *C. clarkii*, *C. santaanae*, and *C. plebeius*.

## Discussion

Phylogenetic incongruence derived from different genes and/or methodologies is problematic for modern systematics [1–3]. However, the complexity of these evolutionary histories can potentially be resolved through phylogenomics, even in the face of reticulation and the phylogenetic incongruence it fosters [7].

In this study, we dissected the disagreements between previously generated mitochondrial and morphological phylogenies of fine-scale sucker by: 1) resolving a nuclear phylogeny for *Catostomus,* and 2) testing for the presence of introgression. In doing so, we invoked several different analytical approaches, and these in turn allowed us to evaluate: A) the effects of gene incongruence on concatenated and multi-species coalescent methods, and B) naïve binning as a method to test for potential effects of concatenation. We then applied D-statistic tests to successfully unravel phylogenetic discord in *Catostomus* and test if convergent evolution or introgression were potential components of its reticulated evolutionary history.

### Effects of introgression on concatenated and MSC phylogenetic analyses

The genus *Catostomus* is comprised of at least 26 species, distributed primarily throughout western North America [70]. Recent phylogenetic evaluations [35, 54] argued for taxonomic revisions, to include: Recognition of four recently described or re-designated species (*C. virescens*, *C. bondi*, *C. lahontan*, and *C. jordani*); confirmation of hybrid origin for two species (*C. columbianus, C. discobolus yarrowi*); and clarification of conflicting phylogenetic hypotheses [35, 38].

The majority of species (i.e., 77%) were evaluated, to include four recently described or re-recognized, and for which support was subsequently derived herein. The few remaining unexamined species are unlikely to change our depiction of relationships, given the number of species employed (per above), and the fact that different methods produced largely congruent topologies, despite the presence of several undetected introgression events. These underscore the phylogenetic robustness of the data, as well as the capacity of the various methodologies to yield a well-supported and consistent phylogeny despite reticulation.

However, topologies produced by concatenated methods (ML and BA) versus multi-species coalescent methods (quartet assembly) did differ at a single node and thus this discord should be explored before we move on to the point of resolving discords with mitochondrial and morphological phylogenies.

The focus of this particular conflict was the placement of taxa within the ‘*discobolus*’ group (i.e., *C. discobolus*, *C. virescens*, *C. plebeius*, *C. santaanae*, and *C. clarkii*, [35]). The concatenated methods strongly supported *C. discobolus* and *C. virescens* as sister to the remainder of the group (Fig. 4a), whereas a MSC method (SVDquartets) instead supported *C. plebeius* (Fig. 4b). The latter was also represented as such in previous morphological [35] and mitochondrial phylogenies [54].

The first consideration with regard to this incongruence is the capacity of the methods to resolve short branches. This gains traction since many of the shortest branches in the phylogeny are in the ‘*discobolus*’ group (FF; Fig. 2). However, this in itself is odd, in that a relatively extensive fossil record supports diversifications during the late Pliocene to Mid-Pleistocene [35], a result consistent with geological events and a fossil-calibrated mitochondrial dataset [54]. However, short branches may also be artifacts of introgression, as identified in studies both empirical [71] and simulated [41].

**Fig. 5:**
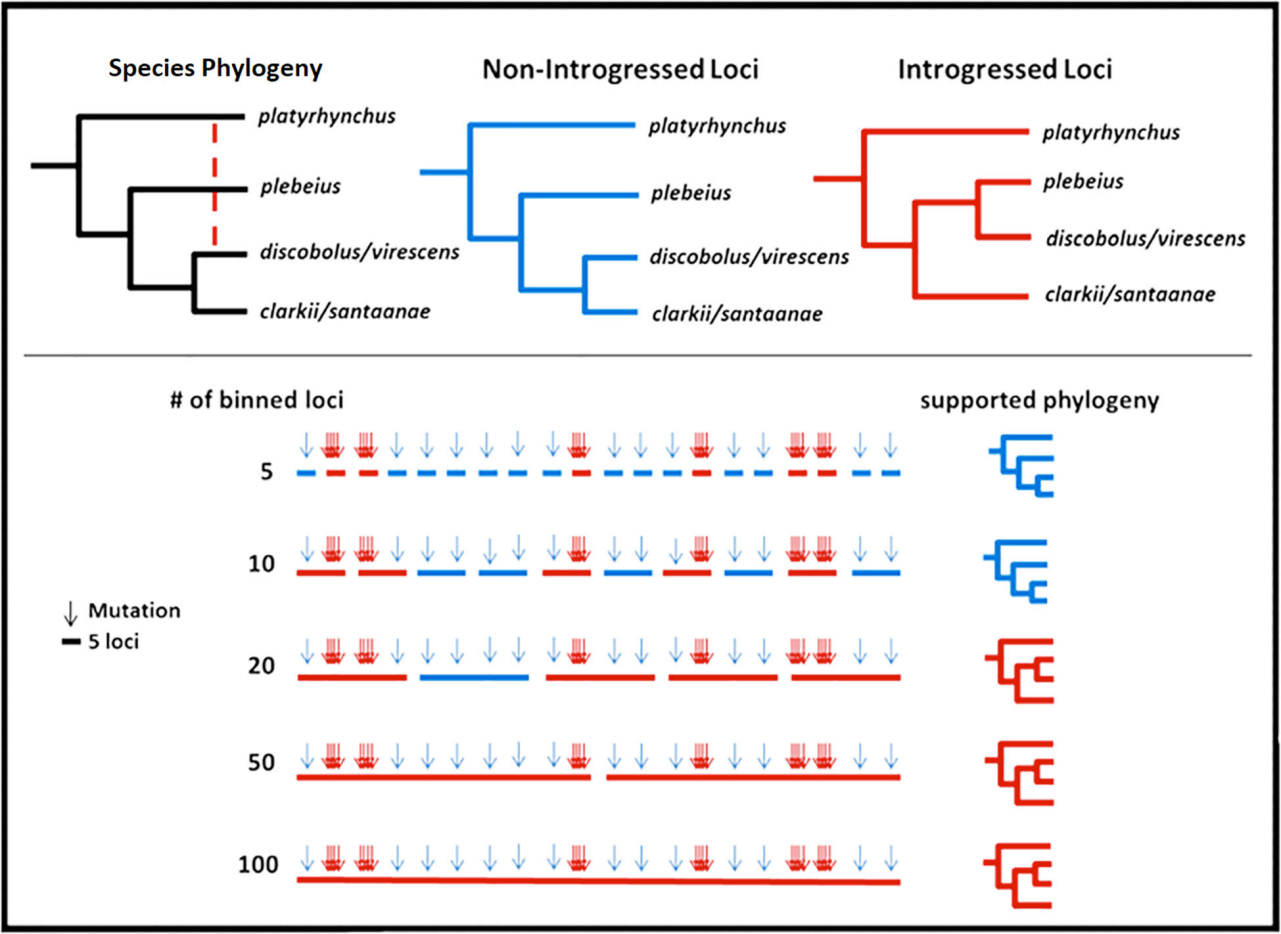
Depiction of the bias on concatenation caused by introgression. Top left phylogeny (black) represents the proposed species phylogeny from morphological [35] and mitochondrial [54] data, with a red dotted line representing significant introgression detected by D-statistic tests. Resulting topologies of non-introgressed (blue) and introgressed (red) loci are shown on top. Below represents the binned loci (solid bars) and corresponding mutations (arrows above loci) that are colored according to the topology supported. Introgressed loci carry more mutations supporting the introgressed topology (red arrows) due to the long divergence between *C. platyrhynchus* and *C. discobolus / C. virescens*. As binning increases, every binned locus that contains both introgressed and non-introgressed loci will reflect the introgressed topology, resulting in more binned loci supporting the introgressed topology as binning increases. Phylogenies to the left of the loci represent the topology supported by ASTRAL for each level of binning with colors corresponding to above

A second potential explanation may indeed be introgression itself, since the ‘*discobolus*’ group is replete with these events (Fig. 2, Table 2). Introgression between *C. platyrhynchus* and *C. discobolus*/*virescens* is particularly problematic in that it occurred between distantly related species. Thus, alleles introgressed from *C. platyrhynchus* into *C. discobolus*/*virescens* may reduce the distance between these species yet increase the distance between *C. discobolus*/*virescens* and the remainder of the ‘*discobolus*’ group. This, in turn, would erroneously place *C. discobolus*/*virescens* outside the ‘*discobolus*’ group. If this is indeed the case, then a smaller percentage of introgressed alleles would be required to contravene the relationship expressed by the majority of loci, thus seriously impacting concatenation (Fig. 5). A more appropriate resolution may thus be provided by the multi-species coalescent (MSC) phylogeny, in that it utilizes only unlinked/ independent SNPs. It would be less impacted by the distance of introgressed alleles (as above) since it reflects relationships found among the majority of loci, whereas results from concatenated method would instead be driven only by the small subset of alleles introgressed among *C. platyrhynchus* and *C. discobolus*/*virescens* (Additional file 3: Table S2).

To further evaluate this argument, we applied a naïve binning approach in which varying amounts of RAD-loci were randomly grouped then subsequently analyzed as “supergenes” by a second MSC method (i.e., Astral). Here, the assumption was that fewer binned RAD-loci should yield results similar to the MSC phylogeny. If discordance is caused by the concatenation of introgressed alleles masking non-introgressed alleles, then binning with a greater number of RAD-loci should shift support to the topology identified by the concatenated methods (Fig. 5). And in fact, this is exactly what we found. Lower levels of binning (≤10 loci) yielded a topology congruent with that of the MSC phylogeny, whereas greater levels (≥15 loci) produced instead a topology that aligned with concatenated methods (Fig. 3). This pattern was also present when loci with little phylogenetic signal (< 2 SNPs per locus) were filtered out. The approach also increased support at lower binning levels, and also the point at which alternative topologies occurred (at 5 instead of 10 binned loci). This pattern was also reflected in the number of trees generated from individual binned loci that supported alternative topologies (Fig. 3). As an aside, no significant introgression between *C. plebeius*, *C. santaanae*, and *C. clarkii* was detected in the D-statistic results, thus eliminating another potential explanation for the erroneous grouping (Table 2, Additional file 3: Table S2).

Our results also paralleled the recent debate between concatenated and MSC methods. One argument [72] indicated that MSC methods relied on unrealistic models that failed to account for gene incongruence, other than from incomplete lineage sorting, and thus were inappropriate for resolving introgressed phylogenies. Concatenation was favored instead, since introgression should be masked, and the phylogeny represented instead by a majority of loci. However, recent studies with simulated data [62, 73] showed both approaches being impacted, even at low levels of introgression, with concatenated methods failing to capture the species tree at reduced levels of gene incongruence, whereas MSC methods required slightly higher levels of gene incongruence to fail. These considerations are also supported herein. However, we suggest this is not an argument for the supremacy of one method over the other, but instead a recognition that multiple approaches are needed and should include multiple lines of evidence including fossil record and morphology. A useful addition to our study would be the application of phylogenetic methods that more appropriately evaluate introgression (i.e. BUCKY), but these are not computationally viable for large datasets such as ours.

### Tests for introgression that resolved phylogenetic discord

We found several statistically significant introgressive events using the D-statistic test, (Fig. 3, Additional file 3: Table S2), and these resolved the conflicts observed between our phylogeny and previous mitochondrial phylogenies (Table 3). They included the following discordant placements found in mitochondrial phylogenies: 1) *X. texanus* as sister to *C. insignis*, 2) *C. platyrhynchus* as sister to *C. discobolus*/*virescens*, 3) some *C. latipinnis* populations placed within *C. insignis*, and 4) some *C. discobolus* populations that fell within *C. plebeius* and *C. clarkii*, (see mitochondrial phylogenies [38, 54, 74]. The only exception was *C. columbianus*, the placement of which matched that recorded in previous mitochondrial phylogenies [38] and with no introgression detected (Table 2). Our results largely confirmed the ‘Introgression Hypothesis’ [35], and reflect the importance of phylogenomic analyses in resolving those instances (as herein) where reticulated evolution has confounded relationships.

**Table 3:** Tests for introgression in Regards to the ‘Introgression Hypothesis’

Introgression Events (Smith et al. 2013)	D-statistic Results
*C. insignis* - *X. texanus*	Confirmed
*C. columbianus* hybrid origin	No introgression detected^a^
*C. platyrhynchus* - *C. discobolus/virescens*	Confirmed
*C. clarkii* - *C. discobolus*	Confirmed
*C. plebius* - *C. d. yarrowi*	Confirmed in one population^b^

D-statistic results in respect to introgression events needed to explain discords with mitochondrial phylogeny detailed by [35] based on their morphology and fossil record work

^a^Tests for *C. columbianus* are a result based off of two samples from nearby sample sites and does not reflect their whole range

^b^Introgression of *C. plebeius only* detected in the Rio Nutria population of *C. d. yarrowi* and not in the other two populations, same result of [69, 90]

Our results also underscored potential dangers inherent in the reliance upon single-gene phylogenies, such as those based on markers from the mitochondrion, in that Dobzhansky-Muller incompatibilities and unipaternal inheritance can lead to a rapid fixation of invasive mitochondria, thus yielding phylogenies discordant with species histories [19]. Despite this admonition, studies aimed at resolving species-relationships and/or developing conservation plans have largely relied upon single mitochondrial or nuclear gene phylogenies [75–77]. Our results underscore the importance of genomic approaches in these situations, and support previous admonitions regarding the sole use of mitochondrial genes in resolving species [78–80].

With regards to *C. columbianus,* our analyses failed to confirm introgression as a confounding factor in its tortuous taxonomy. It was originally described as a component of the genus *Pantosteus* (i.e., *P. columbianus*, Snake River; [81]), then subsequently re-described as a *Catostomus* (i.e., *C. syncheilus*; [82]), and finally as a hybrid lineage with morphological characteristics shared between *C. tahoensis* and an unidentified member of the former *Pantosteus* [35]. Our results instead place *C. columbianus* as sister to *C. tahoensis*, a situation congruent with both the mitochondrial phylogeny. While this also fits with the most recent morphological phylogeny [35], we found no molecular evidence of introgression with any sympatric *Pantosteus* (Fig. 3, Additional file 3: Table S2A), thus arguing against its hypothesized hybrid origin, and supporting instead one of convergent evolution (per [38]). It was the only previously suggested introgression event (per [35]) that was not confirmed (Table 3). However, our diagnosis is based on but two samples (*C. columbianus;* Donner und Blitzen River; Oregon State University Museum), and thus the potential for convergence verses introgression remains open for more substantive testing.

### Introgression and potential endemism in the Colorado River basin

**Fig. 6:**
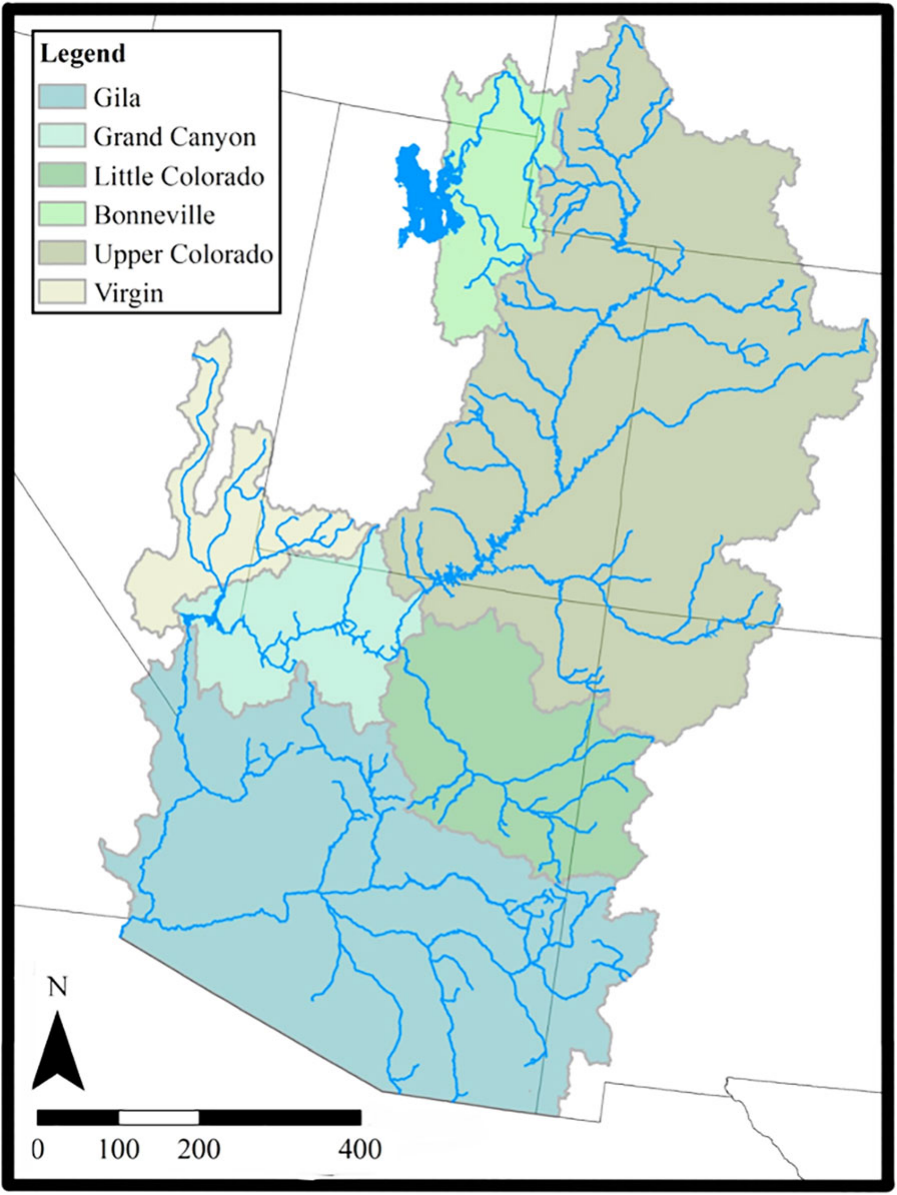
Map of Colorado River Basin and Bonneville Basin

All detected introgression events were between species that occur in the Colorado River and neighboring Bonneville basins (Fig. 6). The foci were three Upper Colorado River Basin species (*C. latipinnis*, *C. discobolus* and *C. platyrhynchus*) that either introgressed among themselves (*C. discobolus* and *C. platyrhynchus*) or with neighboring species (*C. insignis*, *C. clarkii,* and *C. plebeius*). These events were quite difficult to resolve in previous studies [34, 54, 83]. Our phylogenomic approach not only resolved these species but also detected several well supported lineages, each with a history of introgression driven by geographic proximity to our species.

*Catostomus platyrhynchus* is distributed throughout the Bonneville and Upper Colorado River basins (defined as the area above Grand Wash, Fig. 6). Morphologically, *C. platyrhynchus* should be sister to *C. bondi, C. jordani*, and *C. lahontan* from which it recently split [35], as diagnosed herein (Fig. 2). However, in mitochondrial phylogenies, it was indistinguishable from sympatric *C. virescens* and *C. discobolus* [54, 83]. As above, these incongruences were resolved by our tests for introgression (Table 2).

A recent morphological analysis has now separated *C. discobolus* into *C. virescens* (Bonneville Basin/ Upper Snake River) and *C. discobolus* (Upper Colorado River Basin), which were originally described as separate species but later collapsed [35]. In mitochondrial phylogenies, they remain paraphyletic [54, 83], due to the occurrence of several detected introgression events (per this study) that occurred with *C. platyrhynchus*, *C. clarkii,* and *C. plebeius*. Phylogenomic analyses have resolved *C. discobolus* and *C. virescens* as separate sister entities. A similar split across basins was also detected in *C. platyrhynchus,* despite it being listed as a single species.

Two lineages were detected within *C. discobolus,* separating the Little Colorado from conspecifics in the Colorado River Basin (Fig. 2). The Upper Little Colorado River only became isolated from the Colorado River ~20kya by the formation of Grand Falls [84]. However, and despite the recent occurrence of this vicariant event, the Little Colorado River harbors several additional unique species: Little Colorado River Spinedace (*Lepidomeda vittata* [85]), a potentially unique form of *C. latipinnis* (see below, [86, 87]), as well as a unique subspecies of *C. discobolus* (i.e., *C. d. yarrowi*; [88]).

Recently, *C. d. yarrowi* was listed under the Endangered Species Act [89]. It occurs on the Defiance Plateau and in the Zuni River, both of which drain into the Little Colorado River. Yet, our analyses depict each as a discrete clade within a larger paraphyletic group. The paraphyly can be resolved by grouping the remainder of the Little Colorado River with the Defiance Plateau and Zuni River, a consideration potentially supported by the larger caudal fins and more terete shape of individuals within the Little Colorado River, and which also distinguishes *C. d. yarrowi* [87].

A unique form of *C. latipinnis* (i.e., Little Colorado River Sucker) has also been recognized as distinct from the rest of *C. latipinnis* [87], and mentioned as a potential new species replete with a ‘manuscript name’ (*C. sp. “crassicauda;”* [86]). While it does emerge as distinct in our analyses, it also falls within a paraphyletic *C. latipinnis*. Its putative recognition as distinct would necessitate the separation of Virgin River *C. latipinnis* from *C. latipinnis* (sensu *lato*), thus yielding three separate taxa.

Both *C. latipinnis* and *C. discobolus* in the Little Colorado River were introgressed with species in neighboring basins (*C. insignis* in the Lower Colorado River Basin, and *C. plebeius* in the Rio Grande). This differential introgression could account for the phylogenetic split observed with the rest of the Colorado River. In fact, *C. d. yarrowi* was postulated as a hybrid species between *C. discobolus* and *C. plebeius* [90]. However, our results found introgressed alleles from *C. plebeius* in but a single population, i.e., Rio Nutria (Fig. 3, Additional file 3: Table S2G), a result congruent with other studies employing allozymes [91] and single-gene sequencing [69]. Similarly, introgression from *C. insignis* was only detected in one *C. latipinnis* population in the Little Colorado (i.e., Wenima Wildlife Area). Thus, placement of these lineages is unlikely, due either to differential introgression or to hybrid origin.

Morphological support has been offered for the separation of Virgin River *C. latipinnis* [92], but this may also be a result of hybridization with *C. insignis* or *X. texanus* [93].Our D-statistic tests indicated that Virgin River *C. latipinnis* reflects significant introgression with *C. insignis*, thus supporting the hybridization hypothesis. However, a single population was sampled, and thus to verify this situation, additional testing should occur throughout the remainder of the Virgin River.

While *C. discobolus* does not occur in the Virgin River, introgression from *C. discobolus* from the Upper Colorado River was detected in *C. clarkii* from the Virgin River. This provides an interesting pattern in the Virgin River, with introgression occurring among sister pairs from the Upper and Lower Colorado rivers (i.e., *C. latipinnis-C. insignis* and *C. discobolus-C. clarkii).* The extent of this introgression is a worthwhile topic to pursue, in that our samples represent single sites for each species.

## Conclusions

Systematic analyses of many non-model organisms, particularly those that possess a history replete with reticulated evolution, have often been hampered by the discordance between mitochondrial and morphological analyses. However, recent advances in molecular sequencing technology have allowed these phylogenomic histories to be deciphered, with incongruences resolved and unambiguous tests of historical introgression promoted. In this regard, our phylogenomic analyses yielded similar topologies across methods, despite the detection of numerous introgressive events. Yet they also confirmed introgression as a major factor in the discordance found between previously generated mitochondrial and morphology phylogenies.

Additionally, our phylogenomic results differed at but a single node, seemingly due to introgression between distantly related taxa. This result highlights the necessity of utilizing different phylogenetic approaches, such as concatenation and multi-species coalescent methods, particularly when the potential for gene incongruence is elevated. Our results also underscored the need for synthetic analyses that incorporate fossil, morphological, and biogeographic data.

The taxonomic veracity of *Pantosteus* as a monophyletic clade was supported herein [35, 54], as were proposed taxonomic revisions currently being offered, but with the exclusion of *C. columbianus* as a component. Additional morphological and molecular data are needed to further substantiate any subdivisions within *Catostomus* [35], and such analyses must involve the remainder of *Catostomus,* and well as the Lake Suckers, *Chasmistes* and *Deltistes*.

Fine-grained phylogeographic patterns in the Colorado River Basin also warrant additional study, especially with regard to the Virgin and Little Colorado rivers, in that both systems harbor populations with complex histories blurred by recent and historic admixture, yet each is also a focus of conservation concern as well. The results of our analyses promote *Catostomus* as a model system from which the effects of reticulate evolution can be more fully interpreted, and as an exemplar for management and conservation of desert fishes.

## Additional files

Additional file 1: **Figure S1** Presence and absence of loci by individual following guidelines from [108], Presence and absence of loci by individual following guidelines from [94], with loci represented by columns and individuals organized in rows, arranged in the same order as the phylogeny (Fig. 2). Presence of a locus is represented by a black pixel and white represents absence. Presence/absence is split into four lines with the top three containing 3750 loci each and the bottom line consisting of the remaining 2757 loci. (TIF 1199 kb)

Additional file 2: **Table S1** Sample ID, sample locations, number of loci remaining after all filtering steps and percentage of loci out of the total (14,007) for each sample. (DOCX 39 kb)

Additional file 3: **Table S2** Expanded results for Patterson’s D-statistic (per [68]). (DOCX 38 kb)

## Abbreviations

AZ: Arizona
BA: Bayesian
ddRAD: Double digest restriction-site associated
kya: Thousand years ago
ML: Maximum likelihood
MSC: Multispecies coalescent analyses
mtDNA: Mitochondrial deoxynucleic acid
mya: Million years ago
OTU: Operational taxon unit
RAD: Restriction-site associated
SNP: Single nucleotide polymorphism
UT: Utah
WGD: Whole genome duplications
