# Supplementary figures and images for: Unraveling historical introgression and resolving phylogenetic discord within Catostomus (Osteichthys: Catostomidae)
Source: BMC Evol Biol. 2018 Jun 7;18:86. doi: 10.1186/s12862-018-1197-y (PMC5992631; doi:10.1186/s12862-018-1197-y)

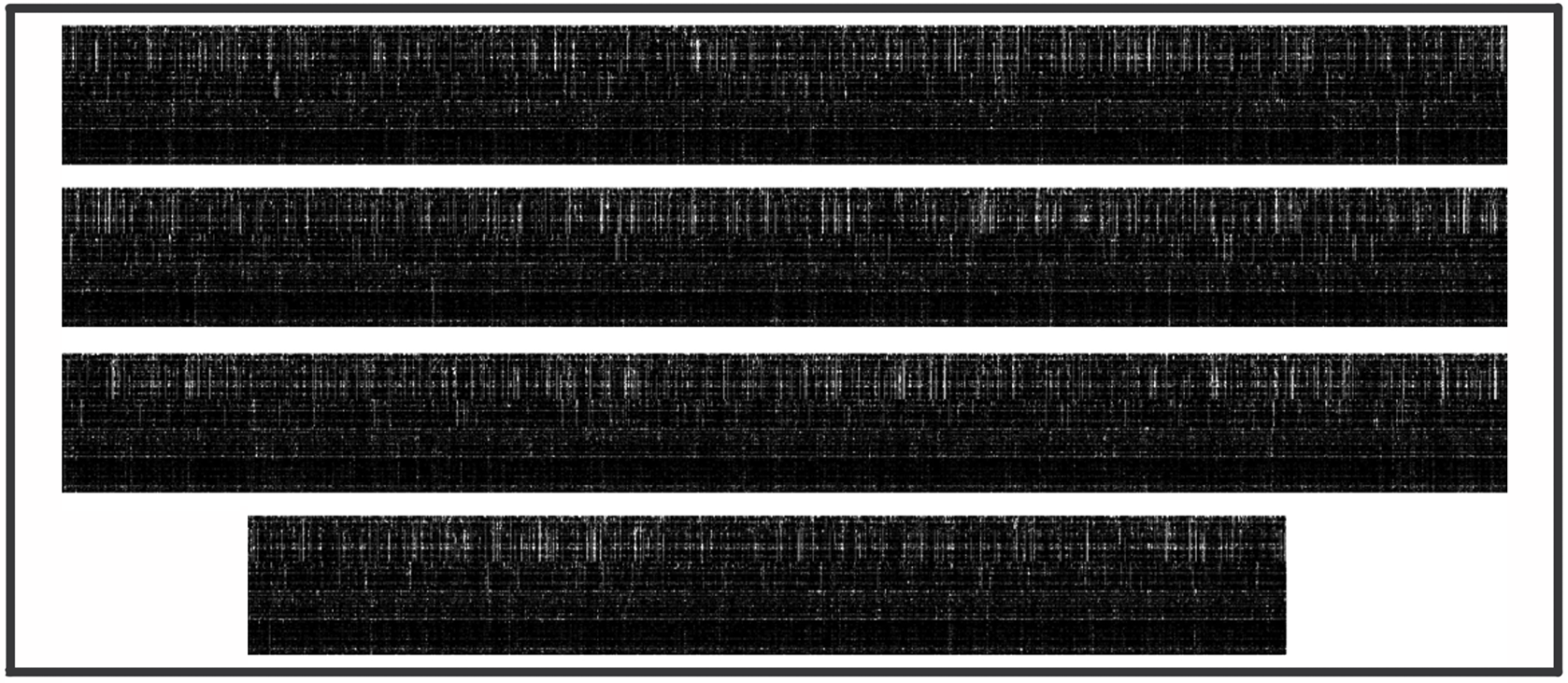

Supplement: Supplementary file 1 — Figure S1 Presence and absence of loci by individual following guidelines from [108], Presence and absence of loci by individual following guidelines from [94], with loci represented by columns and individuals organized in rows, arranged in the same order as the phylogeny (Fig. 2). Presence of a locus is represented by a black pixel and white represents absence. Presence/absence is split into four lines with the top three containing 3750 loci each and the bottom line consisting of the remaining 2757 loci. (TIF 1199 kb) [file 12862_2018_1197_MOESM1_ESM.tif]
