# Supplementary material for: Unraveling historical introgression and resolving phylogenetic discord within Catostomus (Osteichthys: Catostomidae)
Source: BMC Evol Biol. 2018 Jun 7;18:86. doi: 10.1186/s12862-018-1197-y (PMC5992631; doi:10.1186/s12862-018-1197-y)
Supplement: Supplementary file 2 — Table S1 Sample ID, sample locations, number of loci remaining after all filtering steps and percentage of loci out of the total (14,007) for each sample. (DOCX 39 kb) [file 12862_2018_1197_MOESM2_ESM.docx]

**Table S1** Sample ID, sample locations, number of loci remaining after all filtering steps and percentage of loci out of the total (14,007) for each sample

| **Sample ID** | **# of loci** | **% of total** | **Species** | **Major Drainage** | **Sample Site** |
| --- | --- | --- | --- | --- | --- |
| 56hrt006 | 3273 | 23% | ***M. macrolepidotum*** | Missouri River | Heart River |
| 56ott008 | 5308 | 38% | ***M. valenciennesi*** | Red River | Ottertail River |
| 46bsb002 | 7923 | 57% | ***C. catostomus*** | Big Sandy River | Big Sandy River |
| 46bsb004 | 3920 | 28% | ***C. catostomus*** | Big Sandy River | Big Sandy River |
| 46bsb009 | 6494 | 46% | ***C. catostomus*** | Big Sandy River | Big Sandy River |
| 33bvc001 | 9099 | 65% | ***C. commersonii*** | Missouri River | Beaver Creek |
| 33gaf001 | 9052 | 65% | ***C. commersonii*** | Upper Green River | Green River above Flaming Gorge Dam |
| 33gos001 | 8270 | 59% | ***C. commersonii*** | Red River | Goose River |
| 33ill001 | 7255 | 52% | ***C. commersonii*** | Mississippi River | Paisa Creek |
| 33ylp001 | 8597 | 61% | ***C. commersonii*** | Yampa River | Yampa River |
| rimiculus | 3262 | 23% | ***C. rimiculus*** | Rogue River | Rogue River |
| 2osu002 | 7863 | 56% | ***C. microps*** | Goose Lake | Thomas Creek |
| 2ubl001 | 10048 | 72% | ***C. ardens*** | Bear River | Bear Lake |
| 2ubl002 | 9840 | 70% | ***C. ardens*** | Bear River | Bear Lake |
| 2srp001 | 9013 | 64% | ***C. ardens*** | Lower Snake River | Pacific Creek |
| 2srp002 | 10111 | 72% | ***C. ardens*** | Lower Snake River | Pacific Creek |
| 2osu005 | 2806 | 20% | ***C. columbianus*** | Donner und Blitzen River | Booners Creek |
| 2osu004 | 7690 | 55% | ***C. columbianus*** | Donner und Blitzen River | Kiger Creek |
| 2osu001 | 5469 | 39% | ***C. tahoensis*** | Humboldt River | South Fork Reservoir |
| 2tah005 | 8560 | 61% | ***C. tahoensis*** | Truckee River | Truckee River near Reno |
| 2tah007 | 8401 | 60% | ***C. tahoensis*** | Truckee River | Truckee River near Reno |
| 2rbs00X | 8909 | 64% | ***X. texanus*** | San Juan River | Dexter Hatchery |
| 2msb003 | 4152 | 30% | ***X. texanus*** | San Juan River | Dexter Hatchery |
| 2msb004 | 9730 | 69% | ***X. texanus*** | San Juan River | San Juan River |
| 2msb005 | 9657 | 69% | ***X. texanus*** | San Juan River | San Juan River |
| 10smr004 | 8050 | 57% | ***C. insignis*** | Bill Williams River | Santa Maria River |
| 10smr006 | 4074 | 29% | ***C. insignis*** | Bill Williams River | Santa Maria River |
| 10vec004 | 7759 | 55% | ***C. insignis*** | Verde River | Verde River at Croll Property |
| 10ara005 | 9125 | 65% | ***C. insignis*** | Gila River | Aravaipa Creek |
| 10efg011 | 7328 | 52% | ***C. insignis*** | Gila River | East Fork of Gila River |
| 10tul006 | 9001 | 64% | ***C. insignis*** | San Francisco River | Tularosa Creek |
| 10tul011 | 9194 | 66% | ***C. insignis*** | San Francisco River | Tularosa Creek |
| 2bdl001 | 9275 | 66% | ***C. latipinnis*** | Virgin River | Beaver Dam Wash |
| 2bdl002 | 6382 | 46% | ***C. latipinnis*** | Virgin River | Beaver Dam Wash |
| 2bdl003 | 8901 | 64% | ***C. latipinnis*** | Virgin River | Beaver Dam Wash |
| 2bdl004 | 10087 | 72% | ***C. latipinnis*** | Virgin River | Beaver Dam Wash |
| 2bdl005 | 9615 | 69% | ***C. latipinnis*** | Virgin River | Beaver Dam Wash |
| 2bdl006 | 10331 | 74% | ***C. latipinnis*** | Virgin River | Beaver Dam Wash |
| 2bdl011 | 10339 | 74% | ***C. latipinnis*** | Virgin River | Beaver Dam Wash |
| 2bdl019 | 9901 | 71% | ***C. latipinnis*** | Virgin River | Beaver Dam Wash |
| 17ccl001 | 4178 | 30% | ***C. latipinnis*** | Little Colorado River | Chevelon Canyon Lake |
| 17ccl003 | 6391 | 46% | ***C. latipinnis*** | Little Colorado River | Chevelon Canyon Lake |
| 17ccl006 | 8676 | 62% | ***C. latipinnis*** | Little Colorado River | Chevelon Canyon Lake |
| 17svl002 | 8250 | 59% | ***C. latipinnis*** | Little Colorado River | Silver Creek |
| 17svr002 | 4016 | 29% | ***C. latipinnis*** | Little Colorado River | Silver Creek |
| 17wen010 | 7777 | 56% | ***C. latipinnis*** | Little Colorado River | Wenima Wildlife Area |
| 17wen012 | 5331 | 38% | ***C. latipinnis*** | Little Colorado River | Wenima Wildlife Area |
| 17wen013 | 4301 | 31% | ***C. latipinnis*** | Little Colorado River | Wenima Wildlife Area |
| 2bft002 | 8776 | 63% | ***C. latipinnis*** | Upper Green River | Black's Fork |
| 2hfm003 | 10317 | 74% | ***C. latipinnis*** | Upper Green River | Henry's Fork |
| 2bsb004 | 10005 | 71% | ***C. latipinnis*** | Upper Green River | Big Sandy River |
| 2lsc001 | 4656 | 33% | ***C. latipinnis*** | Upper Green River | Little Sandy Creek |
| 2gaf001 | 9555 | 68% | ***C. latipinnis*** | Upper Green River | Green River above Flaming Gorge Dam |
| 2ysv001 | 9230 | 66% | ***C. latipinnis*** | Yampa River | Yampa River |
| 2srr004 | 9814 | 70% | ***C. latipinnis*** | San Rafael River | San Rafael River |
| 2ddq006 | 9477 | 68% | ***C. latipinnis*** | Dirty Devil | Muddy Creek |
| 2sjr006 | 9736 | 70% | ***C. latipinnis*** | San Juan River | San Juan River |
| 2nja003 | 6805 | 49% | ***C. latipinnis*** | San Juan River | Navajo River |
| 2mce002 | 10193 | 73% | ***C. latipinnis*** | Upper Colorado River | McElmo Creek |
| 2kan260 | 10426 | 74% | ***C. latipinnis*** | Colorado River (Grand Canyon) | Kanab Creek |
| 2kan261 | 9570 | 68% | ***C. latipinnis*** | Colorado River (Grand Canyon) | Kanab Creek |
| 2lcr001 | 6548 | 47% | ***C. latipinnis*** | Colorado River (Grand Canyon) | Confluence with Little Colorado River |
| 2shn237 | 10338 | 74% | ***C. latipinnis*** | Colorado River (Grand Canyon) | Shinumo Creek |
| 2shn239 | 10490 | 75% | ***C. latipinnis*** | Colorado River (Grand Canyon) | Shinumo Creek |
| 48bev003 | 6065 | 43% | ***C. jordani*** | Missouri River | Beaver Creek (west of Herron) |
| 48bev004 | 8271 | 59% | ***C. jordani*** | Missouri River | Beaver Creek (west of Herron) |
| 48osu001 | 7081 | 51% | ***C. bondi*** | Willamette River | Willamette River at confl. Santiam River |
| 48osu002 | 7966 | 57% | ***C. bondi*** | Willamette River | Willamette River at river mile 69 |
| 48lah101 | 10740 | 77% | ***C. lahontan*** | Truckee River | Truckee River near Reno |
| 48lah102 | 10425 | 74% | ***C. lahontan*** | Truckee River | Truckee River near Reno |
| 48lah103 | 8019 | 57% | ***C. lahontan*** | Truckee River | Truckee River near Reno |
| 48tah004 | 9173 | 65% | ***C. lahontan*** | Truckee River | Truckee River near Reno |
| 48tah006 | 10540 | 75% | ***C. lahontan*** | Truckee River | Truckee River near Reno |
| 48pwr003 | 10321 | 74% | ***C. platyrhynchus*** | Price River | White River |
| 48pwr004 | 10391 | 74% | ***C. platyrhynchus*** | Price River | White River |
| 48eco001 | 11420 | 82% | ***C. platyrhynchus*** | Weber River | Echo Creek |
| 48gir015 | 10796 | 77% | ***C. platyrhynchus*** | Bear River | Giraffe Creek |
| 48brc001 | 11357 | 81% | ***C. platyrhynchus*** | Lower Snake River | Blackrock Creek |
| 48brc005 | 11000 | 79% | ***C. platyrhynchus*** | Lower Snake River | Blackrock Creek |
| 48mam003 | 10424 | 74% | ***C. platyrhynchus*** | San Rafael River | Mammoth Reservoir |
| 48mam006 | 2861 | 20% | ***C. platyrhynchus*** | San Rafael River | Mammoth Reservoir |
| 48bms005 | 8427 | 60% | ***C. platyrhynchus*** | Locomotive Springs | Bar Mountain Spring |
| 48bms006 | 11526 | 82% | ***C. platyrhynchus*** | Locomotive Springs | Bar Mountain Spring |
| 48hfb001 | 11554 | 82% | ***C. platyrhynchus*** | Upper Green River | Henry's Fork |
| 48lfu021 | 10542 | 75% | ***C. platyrhynchus*** | Upper Green River | Little West Fork |
| 48lsc001 | 11042 | 79% | ***C. platyrhynchus*** | Upper Green River | Little Sandy Creek |
| 48pac007 | 9615 | 69% | ***C. platyrhynchus*** | Upper Green River | Pacific Creek (Big Sandy River) |
| 48nvc002 | 11818 | 84% | ***C. platyrhynchus*** | Upper Green River | North Vermillion Creek |
| 48nvc004 | 10941 | 78% | ***C. platyrhynchus*** | Upper Green River | North Vermillion Creek |
| 48ish011 | 11262 | 80% | ***C. platyrhynchus*** | Upper Green River | Irish Canyon Creek (New Fork River) |
| 48ish003 | 3979 | 28% | ***C. platyrhynchus*** | Upper Green River | Irish Canyon Creek (New Fork River) |
| 48ehc001 | 11888 | 85% | ***C. platyrhynchus*** | Yampa River | Echo Creek |
| 48cuf004 | 12005 | 86% | ***C. platyrhynchus*** | Duchesne River | Current Creek |
| 48drl006 | 8836 | 63% | ***C. platyrhynchus*** | Duchesne River | Lake Fork River |
| 48uin001 | 9635 | 69% | ***C. platyrhynchus*** | Duchesne River | West Fork of Uintah River |
| 48hol008 | 10783 | 77% | ***C. platyrhynchus*** | Upper Colorado River | Hooper Lake |
| 48kel002 | 5102 | 36% | ***C. platyrhynchus*** | Upper Colorado River | Keener Lake |
| 48dil001 | 11341 | 81% | ***C. platyrhynchus*** | Upper Colorado River | Dinkle Lake |
| 48wel001 | 11193 | 80% | ***C. platyrhynchus*** | Upper Colorado River | Weller Lake |
| 19mnc003 | 10508 | 75% | ***C. plebeius*** | Mimbres River | TNC Tract |
| 19mnm015 | 11509 | 82% | ***C. plebeius*** | Mimbres River | NMGF Property |
| 19msb002 | 6631 | 47% | ***C. plebeius*** | Rio Grande | Rio Vallecitos |
| 19msb003 | 2906 | 21% | ***C. plebeius*** | Rio Grande | Rio Tusas |
| 19msb005 | 11422 | 82% | ***C. plebeius*** | Rio Grande | Crestone Creek |
| 19msb006 | 10801 | 77% | ***C. plebeius*** | Rio Grande | Hot Creek |
| 2sas001 | 10580 | 76% | ***C. santaanae*** | Los Angeles River | Big Tujuaga Creek |
| 2sas002 | 3674 | 26% | ***C. santaanae*** | Los Angeles River | Big Tujuaga Creek |
| 2sas003 | 11374 | 81% | ***C. santaanae*** | Los Angeles River | Big Tujuaga Creek |
| 11mvh005 | 12657 | 90% | ***C. clarkii*** | Virgin River | Meadow Valley Wash |
| 11trc001 | 4586 | 33% | ***C. clarkii*** | Bill Williams River | Trout Creek |
| 11trc004 | 9578 | 68% | ***C. clarkii*** | Bill Williams River | Trout Creek |
| 11vpr001 | 9267 | 66% | ***C. clarkii*** | Verde River | Verde River at Perkinsville |
| 11wcc001 | 7461 | 53% | ***C. clarkii*** | Verde River | West Clear Creek |
| 11wfg001 | 11405 | 81% | ***C. clarkii*** | Gila River | West Fork of Gila River |
| 11efg005 | 11695 | 83% | ***C. clarkii*** | Gila River | East Fork of Gila River |
| 11gln002 | 9134 | 65% | ***C. clarkii*** | San Francisco River | San Francisco River at Glenwood |
| 11tul003 | 8365 | 60% | ***C. clarkii*** | San Francisco River | Tularosa Creek |
| 3srp008 | 12719 | 91% | ***C. virescens*** | Lower Snake River | Snake River |
| 3pcu017 | 12863 | 92% | ***C. virescens*** | Lower Snake River | Pole Creek |
| 3brf002 | 6475 | 46% | ***C. virescens*** | Bear River | Smiths Fork |
| 3chk002 | 11508 | 82% | ***C. virescens*** | Weber River | Chalk Creek |
| 3wer006 | 7498 | 54% | ***C. virescens*** | Weber River | Weber River |
| 3bcn002 | 12384 | 88% | ***C. discobolus*** | Defiance Plateau | Bear Canyon Creek |
| 3bcn003 | 12877 | 92% | ***C. discobolus*** | Defiance Plateau | Bear Canyon Creek |
| 3bkw001 | 12600 | 90% | ***C. discobolus*** | Defiance Plateau | Black Soil |
| 3bkw002 | 10161 | 73% | ***C. discobolus*** | Defiance Plateau | Black Soil |
| 3klc001 | 7813 | 56% | ***C. discobolus*** | Defiance Plateau | Kin Li Chee Creek |
| 3klc002 | 12864 | 92% | ***C. discobolus*** | Defiance Plateau | Kin Li Chee Creek |
| 3nut004 | 12300 | 88% | ***C. discobolus*** | Little Colorado River | Nutrioso Creek |
| 3wen001 | 7290 | 52% | ***C. discobolus*** | Little Colorado River | Wenima Wildlife Area |
| 3efl009 | 2979 | 21% | ***C. discobolus*** | Little Colorado River | East Fork of Little Colorado River |
| 3wil001 | 9079 | 65% | ***C. discobolus*** | Little Colorado River | Willow Creek |
| 3wil003 | 4675 | 33% | ***C. discobolus*** | Little Colorado River | Willow Creek |
| 3sic008 | 6299 | 45% | ***C. discobolus*** | Little Colorado River | Silver Creek |
| 3sic015 | 10661 | 76% | ***C. discobolus*** | Little Colorado River | Silver Creek |
| 3rnu024 | 10999 | 79% | ***C. discobolus yarrowii*** | Zuni River | Rio Nutria |
| 3rnu026 | 8427 | 60% | ***C. discobolus yarrowii*** | Zuni River | Rio Nutria |
| 3rnu027 | 11005 | 79% | ***C. discobolus yarrowii*** | Zuni River | Rio Nutria |
| 3rnu028 | 9508 | 68% | ***C. discobolus yarrowii*** | Zuni River | Rio Nutria |
| 3tam004 | 11915 | 85% | ***C. discobolus yarrowii*** | Zuni River | Tampico Springs (Bio-Park Population) |
| 3tam008 | 12137 | 87% | ***C. discobolus yarrowii*** | Zuni River | Tampico Springs (Bio-Park Population) |
| 3tam010 | 10736 | 77% | ***C. discobolus yarrowii*** | Zuni River | Tampico Springs (Bio-Park Population) |
| 3tam025 | 9991 | 71% | ***C. discobolus yarrowii*** | Zuni River | Tampico Springs (Bio-Park Population) |
| 3agr001 | 5336 | 38% | ***C. discobolus yarrowii*** | Zuni River | Agua Remora |
| 3agr002 | 12654 | 90% | ***C. discobolus yarrowii*** | Zuni River | Agua Remora |
| 3agr003 | 11625 | 83% | ***C. discobolus yarrowii*** | Zuni River | Agua Remora |
| 3agr004 | 12839 | 92% | ***C. discobolus yarrowii*** | Zuni River | Agua Remora |
| 3bss002 | 11359 | 81% | ***C. discobolus*** | Upper Green River | Big Sandy River |
| 3lsb007 | 13047 | 93% | ***C. discobolus*** | Upper Green River | Little Sandy Creek |
| 3rir001 | 12105 | 86% | ***C. discobolus*** | Upper Green River | Ringdahl Reservoir (Little Dry Creek) |
| 3bfa001 | 12851 | 92% | ***C. discobolus*** | Upper Green River | Blacks Fork (Anadarko Property) |
| 3hfm001 | 11501 | 82% | ***C. discobolus*** | Upper Green River | Hams Fork |
| 3gbn001 | 11884 | 85% | ***C. discobolus*** | Middle Green River | Green River |
| 3gsm002 | 10627 | 76% | ***C. discobolus*** | Middle Green River | Split Mountain |
| 3cub006 | 7453 | 53% | ***C. discobolus*** | Middle Green River | Cub Creek (Desolation Canyon) |
| 3yam001 | 11062 | 79% | ***C. discobolus*** | Yampa River | Yampa River |
| 3wir010 | 11020 | 79% | ***C. discobolus*** | White River | White River |
| 3prr001 | 12664 | 90% | ***C. discobolus*** | Price River | Price River |
| 3joe004 | 13019 | 93% | ***C. discobolus*** | San Rafael River | Joe's Valley Reservoir |
| 3srr007 | 7279 | 52% | ***C. discobolus*** | San Rafael River | San Rafael River |
| 3ddq001 | 12168 | 87% | ***C. discobolus*** | Dirty Devil | Quitchupah Creek |
| 3mur001 | 9327 | 67% | ***C. discobolus*** | Dirty Devil | Muddy Creek |
| 3esc002 | 12520 | 89% | ***C. discobolus*** | Escalante River | Escalante River |
| 3pin003 | 7016 | 50% | ***C. discobolus*** | Escalante River | Pine Creek |
| 3bkr012 | 10954 | 78% | ***C. discobolus*** | Upper Colorado River | Black Rocks Canyon |
| 3c15003 | 12045 | 86% | ***C. discobolus*** | Upper Colorado River | 15-mil reach Colorado River |
| 3dor004 | 11482 | 82% | ***C. discobolus*** | Upper Colorado River | Dolores River |
| 3wwc009 | 10556 | 75% | ***C. discobolus*** | Upper Colorado River | Westwater Canyon |
| 3man002 | 11872 | 85% | ***C. discobolus*** | San Juan River | San Juan River |
| 3arh010 | 11946 | 85% | ***C. discobolus*** | San Juan River | Arch Canyon |
| 3arh012 | 12106 | 86% | ***C. discobolus*** | San Juan River | Arch Canyon |
| 3nja001 | 13120 | 94% | ***C. discobolus*** | San Juan River | Navajo River |
| 3nja003 | 12967 | 93% | ***C. discobolus*** | San Juan River | Navajo River |
| 3coy004 | 12995 | 93% | ***C. discobolus*** | Canyon de Chelly (Chinle Creek) | Coyote Wash |
| 3cyc001 | 12993 | 93% | ***C. discobolus*** | Canyon de Chelly (Chinle Creek) | Crystal Creek |
| 3tsa002 | 11439 | 82% | ***C. discobolus*** | Canyon de Chelly (Chinle Creek) | Tsaile Creek |
| 3whe002 | 12296 | 88% | ***C. discobolus*** | Canyon de Chelly (Chinle Creek) | Wheatfield Creek |
| 3why022 | 13088 | 93% | ***C. discobolus*** | Canyon de Chelly (Chinle Creek) | Whiskey Creek |
| 3hav043 | 12543 | 90% | ***C. discobolus*** | Colorado River (Grand Canyon) | Havasu Creek |
| 3kan001 | 6103 | 44% | ***C. discobolus*** | Colorado River (Grand Canyon) | Kanab Creek |
| 3lcr016 | 11488 | 82% | ***C. discobolus*** | Colorado River (Grand Canyon) | Confluence with Little Colorado River |
| 3lcr042 | 11402 | 81% | ***C. discobolus*** | Colorado River (Grand Canyon) | Confluence with Little Colorado River |
| 3mat018 | 12170 | 87% | ***C. discobolus*** | Colorado River (Grand Canyon) | Matkatamiba Canyon |
| 3shn006 | 4534 | 32% | ***C. discobolus*** | Colorado River (Grand Canyon) | Shinumo Creek |
| **Average** | **9381** | **67%** |  |  |  |
