# Supplementary material for: Unraveling historical introgression and resolving phylogenetic discord within Catostomus (Osteichthys: Catostomidae)
Source: BMC Evol Biol. 2018 Jun 7;18:86. doi: 10.1186/s12862-018-1197-y (PMC5992631; doi:10.1186/s12862-018-1197-y)
Supplement: Supplementary file 3 — Table S2 Expanded results for Patterson’s D-statistic (per [68]). (DOCX 38 kb) [file 12862_2018_1197_MOESM3_ESM.docx]

**Table S2** Expanded results for Patterson’s D-statistic

| **A** |  |  |  |  |  |  |  |  |  |  |  |
| --- | --- | --- | --- | --- | --- | --- | --- | --- | --- | --- | --- |
| P1 | P2 | P3 | O | D | std(D) | Z | BABA | ABBA | nloci | RangeZ | nSig/ntest |
| THS | BLS | BBS | LNS | 0.20 | 0.48 | 0.40 | 6 | 10 | 1198 | 0 , 2.88 | 0/90 |
| THS | BLS | MTS (CB) | LNS | 0.20 | 0.65 | 0.31 | 4 | 6 | 945 | 0 , 2.73 | 0/36 |
| THS | BLS | MTS (LB) | LNS | 0.19 | 0.56 | 0.34 | 7 | 10 | 1206 | 0 , 2.97 | 0/90 |
| THS | BLS | MTS (BB) | LNS | 0.19 | 0.53 | 0.36 | 7 | 10 | 1273 | 0 , 2.97 | 0/108 |
|  |  |  |  |  |  |  |  |  |  |  |  |
| **B** |  |  |  |  |  |  |  |  |  |  |  |
| P1 | P2 | P3 | O | D | std(D) | Z | BABA | ABBA | nloci | RangeZ | nSig/ntest |
| FMS (UC) | FMS (GC) | SOS | WTS | 0.08 | 0.27 | 0.30 | 13 | 15 | 4586 | 0 , 2.96 | 0/360 |
| FMS (UC) | **FMS (VR)** | **SOS** | WTS | **0.64** | 0.09 | **7.43** | 21 | 98 | 4706 | **4.76 , 12.69** | **420/420** |
| FMS (GC) | **FMS (VR)** | **SOS** | WTS | **0.64** | 0.07 | **9.01** | 18 | 84 | 4604 | **4.74 , 11.02** | **420/420** |
| FMS (LC) | **FMS (VR)** | **SOS** | WTS | **0.63** | 0.11 | **5.80** | 18 | 77 | 3340 | 3.51 , **11.68** | **338/350** |
| FMS (wen) | FMS (VR) | SOS | WTS | 0.14 | 0.21 | 0.68 | 43 | 57 | 3280 | 0.09 , **4.51** | **35/210** |
| FMS (LC) | FMS (UC) | SOS | WTS | 0.02 | 0.15 | 0.12 | 20 | 21 | 3295 | 0 , 1.54 | 0/300 |
| FMS (LC) | FMS (GC) | SOS | WTS | 0.08 | 0.18 | 0.42 | 20 | 24 | 3208 | 0 , 2.2 | 0/300 |
| FMS (UC) | **FMS (wen)** | **SOS** | WTS | **0.48** | 0.10 | **4.56** | 17 | 49 | 3213 | 0.37 , **7.19** | **129/180** |
| FMS (GC) | **FMS (wen)** | **SOS** | WTS | **0.49** | 0.12 | **4.08** | 17 | 50 | 3126 | 0.21 , **7.43** | **126/180** |
|  |  |  |  |  |  |  |  |  |  |  |  |
| **C** |  |  |  |  |  |  |  |  |  |  |  |
| P1 | P2 | P3 | O | D | std(D) | Z | BABA | ABBA | nloci | RangeZ | nSig/ntest |
| FMS (UC) | **SOS** | **RBS** | WTS | **0.54** | 0.05 | **10.16** | 44 | 149 | 3823 | **4.38 , 10.81** | **240/240** |
| FMS (GC) | **SOS** | **RBS** | WTS | **0.56** | 0.06 | **9.10** | 40 | 141 | 3745 | **4.10 . 11.00** | **240/240** |
| FMS (VR) | **SOS** | **RBS** | WTS | **0.49** | 0.05 | **9.80** | 40 | 116 | 3871 | 3.76 , **8.31** | **276/280** |
| FMS (LC) | **SOS** | **RBS** | WTS | **0.53** | 0.08 | **6.41** | 32 | 105 | 2711 | 2.92 , **10.47** | **184/200** |
| FMS (wen) | **SOS** | **RBS** | WTS | **0.54** | 0.10 | **5.33** | 20 | 66 | 2672 | 2.73 , **8.43** | **99/120** |
|  |  |  |  |  |  |  |  |  |  |  |  |
| **D** |  |  |  |  |  |  |  |  |  |  |  |
| P1 | P2 | P3 | O | D | std(D) | Z | BABA | ABBA | nloci | RangeZ | nSig/ntest |
| FMS (GC) | FMS (UC) | RBS | WTS | 0.00 | 0.31 | 0.01 | 12 | 12 | 4259 | 0 , 2.69 | 0/288 |
| FMS (UC) | FMS (LC) | RBS | WTS | 0.06 | 0.24 | 0.27 | 12 | 14 | 3019 | 0 , 2.00 | 0/240 |
| FMS (GC) | FMS (LC) | RBS | WTS | 0.10 | 0.26 | 0.38 | 13 | 16 | 2944 | 0 , 2.48 | 0/240 |
| FMS (UC) | FMS (wen) | RBS | WTS | 0.18 | 0.23 | 0.76 | 13 | 18 | 2891 | 0 , 2.45 | 0/144 |
| FMS (GC) | FMS (wen) | RBS | WTS | 0.13 | 0.25 | 0.51 | 14 | 18 | 2817 | 0.13 , 2.39 | 0/144 |
| FMS (UC) | FMS (VR) | RBS | WTS | 0.44 | 0.14 | 3.10 | 18 | 46 | 4337 | 1.04, **6.01** | **112/336** |
| FMS (GC) | FMS (VR) | RBS | WTS | 0.43 | 0.15 | 2.90 | 16 | 39 | 4249 | 0.98 , **5.57** | **81/336** |
| FMS (LC) | FMS (VR) | RBS | WTS | 0.42 | 0.12 | 3.33 | 15 | 37 | 3013 | 0.91 , **5.05** | **63/280** |
| FMS (wen) | FMS (VR) | RBS | WTS | 0.25 | 0.17 | 1.45 | 17 | 28 | 2911 | 0.18 , 3.26 | 0/168 |
|  |  |  |  |  |  |  |  |  |  |  |  |
| **E** |  |  |  |  |  |  |  |  |  |  |  |
| P1 | P2 | P3 | O | D | std(D) | Z | BABA | ABBA | nloci | RangeZ | nSig/ntest |
| BBS | **BHS (UC)** | **DES (GI)** | MTS (MR) | **0.69** | 0.07 | **9.95** | 34 | 186 | 3761 | **8.01 , 18.35** | **250/250** |
| BBS | **BHS (GC)** | **DES (GI)** | MTS (MR) | **0.45** | 0.09 | **5.00** | 43 | 116 | 3189 | **4.56 , 17.96** | **300/300** |
| BBS | **BHS (def)** | **DES (GI)** | MTS (MR) | **0.40** | 0.09 | **4.72** | 52 | 123 | 3598 | 2.53 , **7.53** | **245/300** |
| BBS | **BHS (tam)** | **DES (GI)** | MTS (MR) | **0.36** | 0.08 | **4.52** | 50 | 106 | 3599 | 3.13 , **6.05** | **122/200** |
| BBS | **BHS (agr)** | **DES (GI)** | MTS (MR) | **0.37** | 0.08 | **4.80** | 49 | 109 | 3315 | 3.14 , **6.15** | **148/200** |
| BBS | **BHS (rnu)** | **DES (GI)** | MTS (MR) | **0.43** | 0.08 | **5.64** | 43 | 108 | 3325 | 3.61 , **6.90** | **184/200** |
| BBS | **BHS (LC)** | **DES (GI)** | MTS (MR) | **0.44** | 0.10 | **4.58** | 41 | 104 | 3213 | 2.72 , **5.77** | **68/100** |
| BBS | BHS (wil) | DES (GI) | MTS (MR) | 0.13 | 0.11 | 1.21 | 38 | 49 | 2361 | 0.12 , 2.66 | 0/100 |
| BBS | BHS (sic) | DES (GI) | MTS (MR) | 0.13 | 0.12 | 1.13 | 56 | 72 | 2877 | 0.14 , 2.96 | 0/100 |
| SAS | **DES (VR)** | **BHS (UC)** | MTS (MR) | **0.43** | 0.09 | **4.63** | 41 | 104 | 3853 | **3.99 , 7.08** | **30/30** |
| SAS | DES (BW) | BHS (UC) | MTS (MR) | 0.18 | 0.15 | 1.20 | 25 | 37 | 2317 | 0.21 , 2.66 | 0/60 |
| SAS | DES (GI) | BHS (UC) | MTS (MR) | 0.28 | 0.10 | 2.70 | 25 | 44 | 3188 | 0.89 , 3.13 | 0/150 |
|  |  |  |  |  |  |  |  |  |  |  |  |
| **F** |  |  |  |  |  |  |  |  |  |  |  |
| P1 | P2 | P3 | O | D | std(D) | Z | BABA | ABBA | nloci | RangeZ | nSig/ntest |
| RGS | **BBS** | **MTS (BB)** | LNS | **0.36** | 0.09 | **4.12** | 24 | 51 | 2397 | 2.22 , **5.21** | **384/540** |
| RGS | **BHS (UC)** | **MTS (BB)** | LNS | **0.44** | 0.08 | **5.32** | 22 | 57 | 2790 | 2.58 , **6.60** | **443/540** |
| RGS | **BHS (GC)** | **MTS (BB)** | LNS | **0.43** | 0.09 | **4.98** | 19 | 49 | 2285 | 2.17 , **6.16** | **475/648** |
| RGS | **BBS** | **MTS (CR)** | LNS | **0.40** | 0.09 | **4.54** | 21 | 49 | 2454 | 2.24 , **5.63** | **434/540** |
| RGS | **BHS (UC)** | **MTS (CR)** | LNS | **0.49** | 0.09 | **5.56** | 21 | 60 | 2863 | 2.87 , **9.28** | **497/540** |
| RGS | **BHS (GC)** | **MTS (CR)** | LNS | **0.48** | 0.10 | **5.02** | 18 | 51 | 2347 | 2.32 , **7.57** | **507/648** |
| MTS (MR) | **MTS (BB)** | **BBS** | LNS | **0.38** | 0.09 | **4.00** | 25 | 55 | 2222 | 2.71 , **6.30** | **156/180** |
| MTS (MR) | **MTS (BB)** | **BHS (UC)** | LNS | **0.32** | 0.08 | **4.07** | 30 | 58 | 2565 | 2.26 , **5.58** | **140/180** |
| MTS (MR) | **MTS (BB)** | **BHS (GC)** | LNS | **0.35** | 0.09 | **4.05** | 22 | 47 | 2105 | 2.21 , **5.63** | **163/216** |
| MTS (MR) | **MTS (CR)** | **BBS** | LNS | **0.43** | 0.09 | **4.68** | 23 | 58 | 2250 | 3.00 , **9.65** | **171/180** |
| MTS (MR) | **MTS (CR)** | **BHS (UC)** | LNS | **0.45** | 0.09 | **4.83** | 25 | 67 | 2608 | 2.83 , **7.66** | **164/180** |
| MTS (MR) | **MTS (CR)** | **BHS (GC)** | LNS | **0.49** | 0.10 | **4.98** | 19 | 55 | 2139 | 2.88 , **11.72** | **202/216** |
| BHS (UC) | BBS | MTS (BB) | LNS | 0.02 | 0.15 | 0.15 | 26 | 27 | 3182 | 0 , 1.78 | 0/450 |
| BHS (GC) | BBS | MTS (BB) | LNS | 0.01 | 0.21 | 0.03 | 23 | 22 | 2593 | 0 , 2.06 | 0/540 |
| BBS | BHS (UC) | MTS (CR) | LNS | 0.04 | 0.17 | 0.21 | 25 | 27 | 3263 | 0 , 2.01 | 0/450 |
| BBS | BHS (GC) | MTS (CR) | LNS | 0.07 | 0.21 | 0.34 | 21 | 24 | 2664 | 0 , 2.41 | 0/540 |
|  |  |  |  |  |  |  |  |  |  |  |  |
| **G** |  |  |  |  |  |  |  |  |  |  |  |
| P1 | P2 | P3 | O | D | std(D) | Z | BABA | ABBA | nloci | RangeZ | nSig/ntest |
| BHS (agr) | **BHS (rnu)** | **RGS** | LNS | **0.95** | 0.05 | **20.04** | 3 | 114 | 3127 | **7.27 , 62.25** | **192/192** |
| BHS (tam) | **BHS (rnu)** | **RGS** | LNS | **0.94** | 0.04 | **21.56** | 4 | 132 | 3567 | **10.51 , 63.19** | **192/192** |
| BHS (LC) | **BHS (rnu)** | **RGS** | LNS | **0.84** | 0.10 | **8.27** | 12 | 133 | 2907 | **4.35 , 36.52** | **96/96** |
| BHS (wil) | **BHS (rnu)** | **RGS** | LNS | **0.83** | 0.09 | **9.31** | 9 | 96 | 2141 | **4.64 , 26.64** | **96/96** |
| BHS (sic) | **BHS (rnu)** | **RGS** | LNS | **0.85** | 0.06 | **14.65** | 10 | 125 | 2657 | **6.24 , 31.60** | **96/96** |
| BHS (def) | **BHS (rnu)** | **RGS** | LNS | **0.78** | 0.06 | **13.44** | 21 | 174 | 3325 | **8.97 , 23.64** | **288/288** |
| BHS (GC) | **BHS (rnu)** | **RGS** | LNS | **0.73** | 0.08 | **8.93** | 26 | 164 | 2904 | **4.72 , 18.77** | **288/288** |
| BHS (agr) | BHS (tam) | RGS | MTS (MR) | 0.10 | 0.52 | 0.19 | 5 | 6 | 2511 | 0.06 , 0.11 | 0/64 |
| BHS (LC) | BHS (agr) | RGS | MTS (MR) | 0.11 | 0.29 | 0.37 | 14 | 17 | 1772 | 0.14 , 1.17 | 0/128 |
| BHS (LC) | BHS (tam) | RGS | MTS (MR) | 0.09 | 0.31 | 0.29 | 17 | 21 | 2012 | 0.01 , 0.91 | 0/128 |
|  |  |  |  |  |  |  |  |  |  |  |  |
| **H** |  |  |  |  |  |  |  |  |  |  |  |
| P1 | P2 | P3 | O | D | std(D) | Z | BABA | ABBA | nloci | RangeZ | nSig/ntest |
| DES (GI) | SAS | RGS | MTS (MR) | 0.15 | 0.20 | 0.75 | 17 | 23 | 2381 | 0.18 , 2.51 | 0/180 |
| DES (VR) | SAS | RGS | MTS (MR) | 0.22 | 0.20 | 1.10 | 24 | 38 | 2845 | 0.23 , 3.52 | 0/36 |
| DES (BW) | SAS | RGS | MTS (MR) | 0.06 | 0.24 | 0.23 | 17 | 19 | 1745 | 0 , 2.32 | 0/72 |
|  |  |  |  |  |  |  |  |  |  |  |  |

Tests with significant Z-scores are in bold with the species involved with introgression also in bold. Range of Z-scores for each set of tests (RangeZ) and the number of significant tests out of the total number of tests (nSig/ntest) are also reported, as is the overall Z-score (Z), average number of alternatively discordant loci (BABA and ABBA) and the average number of loci per test (nloci). Taxa used included one outgroup (O) two sister taxa (P1 and P2) and one taxa outside of P1 and P2 (P3). Positive D-statistics (D) represents an excess of loci supporting ABBA verses BABA topologies indicating potential introgression between taxa P2 and P3. Abbreviations are as follows: THS=Tahoe Sucker, BLS=Bridgelip Sucker, BBS=Bonneville Bluehead Sucker, BHS=Bluehead Sucker (split into UC=Upper Colorado River Basin, GC=Grand Canyon, def=Defiance Plateau, tam=Tampico Springs, agr=Agra Remora, rnu=Rio Nutria, LC=Upper Little Colorado, wil=Willow Creek, and sil=Silver Creek), MTS=Mountain Sucker (split into MR=Missouri River, LB=Lahontan, CB=Columbia River Basin, BB=Bonneville, CR=Colorado River), LNS=Longnose Sucker, SOS=Sonora Sucker, FMS=Flannelmouth Sucker (split into GC=Grand Canyon, UC=Upper Colorado River Basin, LC=Little Colorado without Wenima Wildlife Area, wen=Wenima Wildlife Area, VR=Virgin River), DES=Desert Sucker (split into VR=Virgin River, BW=Bill Williams River, GI=Gila River Basin), WTS=White Sucker, RBS=Razorback Sucker, SAS=Santa Anna Sucker, RGS=Rio Grande Sucker. Tests are split into tests for introgression between (A) Tahoe Sucker x *Pantosteus*, (B) Flannelmouth Sucker x Sonora Sucker, (C) Sonora Sucker x Razorback Sucker, (D) Flannelmouth Sucker x Razorback Sucker, (E) Bluehead Sucker x Desert Sucker, (F) Bluehead Sucker x Mountain Sucker, (G) Zuni Bluehead Sucker x Rio Grande Sucker, and (H) Desert Sucker x Rio Grande Sucker.
